# Kinked silicon nanowires-enabled interweaving electrode configuration for lithium-ion batteries

**DOI:** 10.1038/s41598-018-28108-3

**Published:** 2018-06-28

**Authors:** Georgiana Sandu, Michael Coulombier, Vishank Kumar, Hailu G. Kassa, Ionel Avram, Ran Ye, Antoine Stopin, Davide Bonifazi, Jean-François Gohy, Philippe Leclère, Xavier Gonze, Thomas Pardoen, Alexandru Vlad, Sorin Melinte

**Affiliations:** 1 Institute of Information and Communication Technologies, Electronics and Applied Mathematics, Université catholique de Louvain, 1348 Louvain-la-Neuve, Belgium; 2 Institute of Mechanics, Materials, and Civil Engineering, Université catholique de Louvain, 1348 Louvain-la-Neuve, Belgium; 3 Institute of Condensed Matter and Nanosciences, Université catholique de Louvain, 1348 Louvain-la-Neuve, Belgium; 4 Laboratory for Chemistry of Novel Materials, Center for Innovation and Research in Materials and Polymers, University of Mons, 7000 Mons, Belgium; 5 School of Chemistry, Cardiff University, Park Place, Main Building, Cardiff, CF10 3AT United Kingdom; 6 Department of Chemistry, University of Namur, Rue de Bruxelles 61, 5000 Namur, Belgium

## Abstract

A tri-dimensional interweaving kinked silicon nanowires (k-SiNWs) assembly, with a Ni current collector co-integrated, is evaluated as electrode configuration for lithium ion batteries. The large-scale fabrication of k-SiNWs is based on a procedure for continuous metal assisted chemical etching of Si, supported by a chemical peeling step that enables the reuse of the Si substrate. The kinks are triggered by a simple, repetitive etch-quench sequence in a HF and H_2_O_2_-based etchant. We find that the inter-locking frameworks of k-SiNWs and multi-walled carbon nanotubes exhibit beneficial mechanical properties with a foam-like behavior amplified by the kinks and a suitable porosity for a minimal electrode deformation upon Li insertion. In addition, ionic liquid electrolyte systems associated with the integrated Ni current collector repress the detrimental effects related to the Si-Li alloying reaction, enabling high cycling stability with 80% capacity retention (1695 mAh/g_Si_) after 100 cycles. Areal capacities of 2.42 mAh/cm^2^ (1276 mAh/g_electrode_) can be achieved at the maximum evaluated thickness (corresponding to 1.3 mg_Si_/cm^2^). This work emphasizes the versatility of the metal assisted chemical etching for the synthesis of advanced Si nanostructures for high performance lithium ion battery electrodes.

## Introduction

Albeit extensive theoretical and experimental research on new electrode materials and architectures, there has been moderate progress in conceiving a lithium-ion battery (LIB) able to meet the demanding requirements for high-energy density, long life, safety and cost^1,2^. Guided by these requisites, the quest for next-generation electrode materials for rechargeable LIBs that respond to both industrial and tight environmental constraints has been intensified.

Silicon has been extolled as one of the most appealing high-energy anode material for LIBs. Due to its low working voltage and high specific capacity, Si has animated a plethora of studies directed towards the development of a high capacity Si-based anode^3–7^. The reaction kinetics has been found to be orientation-depended with <110> direction being the fastest, explaining the swelling anisotropy observed in lithiated-Si that sources fractures in the regions with high stress concentrations^8^. In addition, the loss of the Si crystallinity affects its electrical and mechanical properties. To make up for these shortcomings, new designs of Si anodes such as engineered composites and complex architectures are emerging and with that, increased complexity of the electrode manufacturing^9–11^. Indeed, nanostructured Si can partially temper these failure mechanisms by relaxing the strain developed during Li insertion thus, avoiding the onset of fractures^6,12^. However, the high specific surface of nanostructured Si in contact with the electrolyte promotes the generation of excessive solid-electrolyte-interface (SEI). In addition, the high stress-strain cycling conditions weaken and ultimately break the SEI, unwrapping Si and exposing it to the electrolyte^13^. This recursive SEI formation eventually consumes Si as well as the electrolyte, causing battery failure. Several solutions have been proposed for the emergence of a steady SEI layer on nanostructured Si. Among the first in this direction were (i) functional coatings including metal, carbon or oxides and (ii) impermeable binders such as sodium alginate or carboxyl methyl cellulose. These solutions have found some success in enhancing the cycling life of Si-based electrodes by isolating Si from the electrolyte thus preventing the excess apparition of SEI^14–17^. For instance, functional surface coatings reduce the swelling anisotropy by buffering the volumetric expansion through a non-uniform deformation of the coating, preventing particle isolation^18^. Associated to this, electrically active coatings can enhance the current collection efficiency eliminating the necessity for conductive additives. Also, electrolyte additives such as fluoroethylene carbonate (FEC) or vinylene carbonate (VC) help stabilizing the SEI by preventing further parasitic decomposition reactions^19,20^. Similarly, room temperature ionic liquids (IL) have been found to sustain the formation of a steady SEI composed of small inorganic compounds associated with fast release of F ions^21^. To address the aforementioned Si issues and to enable industrial adoption, a suitable Si-based anode must rely on nano-engineered architectures that can avoid the lithiation-induced cracking and can accommodate the volume changes, while sustaining the scalability and the simplicity required for commercial adoption.

Metal-assisted chemical etching is a versatile, inexpensive tool for the large-scale synthesis of vertically-aligned silicon nanowires (SiNWs) allowing for a precise tuning of diameter and length^22,23^. This top-down processing requires complementary procedures to separate the SiNWs from their parent Si substrate and to allow full exploitation of their properties. Such approaches rely either on mechanical peeling or require subsequent processing such as polymer embedding or metal contacts definition in the case of electro-assisted methods^24–26^. While each method has its particular merit depending on application, a universal solution that assists the extraction of etched SiNWs is still lacking.

Herein we report a continuous synthesis of kinked SiNWs (k-SiNWs) by metal assisted chemical etching sustained by chemical peeling that facilitates the extraction of the k-SiNWs and allows for the reuse of the Si substrate. These nanostructures associated with multi-walled carbon nanotubes (MWCNTs) in a three-dimensional interconnected self-standing assembly are evaluated as lithium ion battery electrodes. We particularly emphasize: (i) the simplicity and availability of the k-SiNWs synthesis, (ii) the mechanical properties of the electrode and, related to that, its porosity and viability as Si-based anode, (iii) the electrochemical performance of the k-SiNWs vs. SiNWs-based assemblies and (iv) the improved behavior through conformal Ni coating passivation of the k-SiNWs-based assemblies. We find that k-SiNWs, when used as active material in a lithium ion battery, show major benefits considering the simplicity of the proposed synthesis, the particular k-SiNWs morphology as well as the favorable association of mechanical and electrochemical properties.

## Continuous synthesis of k-SiNWs sustained by chemical peeling

**Figure 1:**
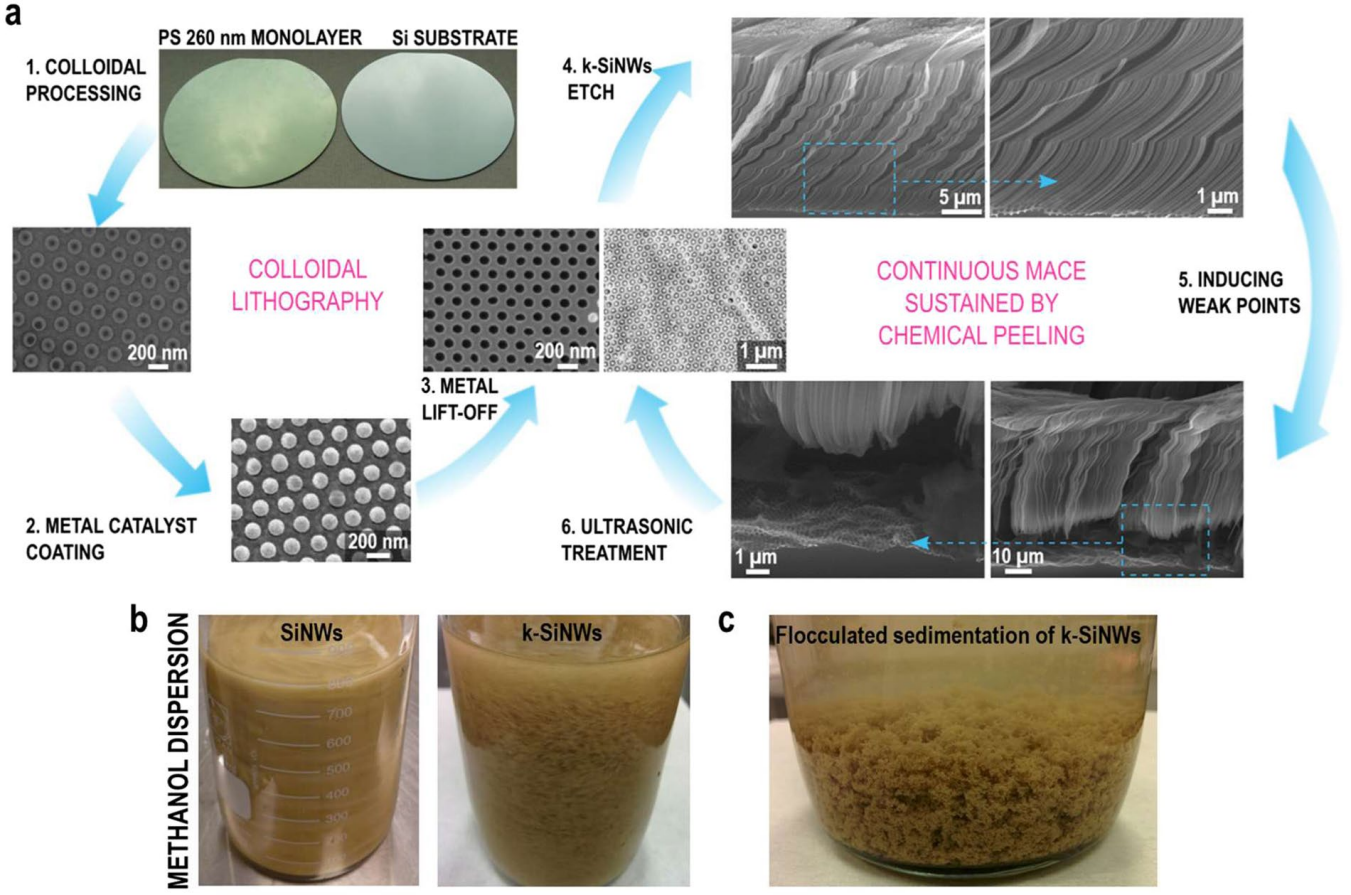
Kinked SiNWs synthesis and processing. Continuous synthesis of k-SiNWs sustained by chemical peeling (**a**). The first step is the nanopatterning of the metal catalyst mask, conveniently achieved by colloidal lithography with polystyrene spheres. Their diameter can be customized by reactive ion etching (1). The holey Au mask can be obtained after the coating of the patterned Si substrate with a thin film (2) and the subsequent removal of the polystyrene spheres (3). The main fabrication step involves etching in a solution containing HF and H_2_O_2_. The etching parameters are favorable for non-<100> etching directions. Interrupting the etching at certain points induces changes in the etching direction and triggers the formation of kinks between adjacent segments (4). When the desired length is achieved, the etching conditions are again modified to favor the presence of a porous segment at the base of the k-SiNWs (5). This porous segment facilitates the separation of k-SiNWs from their Si substrate with a short ultrasonic treatment, leaving the metallic mask intact and ready for a new etching sequence (6). Dispersion of SiNWs vs. k-SiNWs in methanol (**b**). Optical image of the flocculated sedimentation of k-SiNWs (**c**).

Figure 1a resumes the main steps required for the continuous synthesis of k-SiNWs sustained by chemical peeling. Following a typical metal-assisted chemical etching sequence for the fabrication of SiNWs, a Si substrate masked with a holey-patterned catalyst is submitted to an etchant comprising HF and H_2_O_2_. Nanosphere lithography is used to structure the Au catalyst^22,27^, that accommodates an ordered array of nanoholes with 120 nm in diameter and 140 nm spacing (Supplementary Information, Materials and Methods). Under the current understanding, the advancement of the etching front depends on two successive processes: holes generation by H_2_O_2_ decomposition at the metal-Si interface followed by the holes’ injection into the Si valence band. The HF subsequently removes the oxidized Si. As such, the morphology of the etched SiNWs is governed by the downward movement of the metal catalyst mask. According to the back-bond theory, the preferred crystallographic direction along which metal assisted chemical etching proceeds is <100>, regardless of the Si wafer orientation^28–32^. In this study, the generation of k-SiNWs requires two consecutive etching steps of the *p*-type <100> Si substrate in solutions with a different ratio ε of molar concentrations for HF and H_2_O_2_ (ε = [HF]/[H_2_O_2_]) using ethanol as co-solvent. The etching was performed at room temperature, under daylight illumination and with no stirring. The first etching step requires the immersion of the specimen into an etchant containing 9.3 M HF and 1.6 M H_2_O_2_ (ε = 5.8) for 5 minutes. Etching under these conditions is important for the formation of a porous layer under the catalyst layer that subsequently mediates the mass transport of the reactants^33,34^. Furthermore, porosification has been assimilated to a weakening of the back-bonds of the Si’s surface atoms influencing the etching directions^35^. Details of the Si etching under these conditions are presented in Figure S1. The second etching step is carried out in a bath composed of 14 M HF and 1.3 M H_2_O_2_ (ε = 10.7). Under these conditions, the etching follows non-<100> directions (Figures S1 and S2). It was reported that at high ε, the amount of generated holes increases and can polarize more Si back-bonds resulting in non-<100> etching^31,36^. To generate the high aspect ratio k-SiNWs, the etching was quenched every 5 minutes by immersing the Si substrate in methanol. After 1 minute rinse in methanol, the Si substrate is reinserted into the etchant solution for the etching of a new segment. Interrupting the reaction limits the availability of the etchants and helps creating controlled kinks. This etch-quench sequence can be repeated until the desired k-SiNWs length is achieved (Fig. 1).

Overall, the k-SiNWs synthesis for this work requires 6 repetitions of an etch-quench sequence of 5 minutes and, respectively, 1 minute. In addition, adjustments of this scheme allow for the generation of customized Si nanostructures. The length of the etched segment can be varied by altering the etching time, as detailed in Figure S3. This ultimately impacts the k-SiNW’s morphology resulting in particular Si nanostructures that can be tailored to suit specific applications. Next, for the chemical peeling, etching in a solution containing 4.6 M HF and 8.3 M H_2_O_2_ (ε = 0.56) favors the etching of a highly porous segment (Figure S1)^37^. This sacrificial porous segment facilitates the separation of the k-SiNWs from the Si substrate with a short ultrasonic treatment. While other reported techniques pivot around the porosity-mediated separation, the proposed procedure enables fast k-SiNWs extraction as integrated step in metal assisted chemical etching of Si. Furthermore, mask-induced processing defects present on the Si substrate will resist the ultrasonic treatment as the high oxidant concentration is expected to affect only k-SiNWs. This extraction selectivity is a great advantage over other methods such as mechanical peeling that cannot discriminate between the etched nanostructures and defects. After this step, the metal mask remains intact and can be reused for a new etching sequence. The thickness of the Si substrate is the only limitation for this continuous etching scheme. Figure S2 presents a detailed analysis of the etching of a reused Si substrate. A similar continuous etching scheme can be adapted for the continuous etching and extraction of vertical SiNWs (Figure S4). Interestingly, in methanol the SiNWs and k-SiNWs behave differently, as shown in the optical images from Fig. 1b. The SiNWs in methanol, under agitation, have the tendency to align and form domains similar with liquid crystals, while the k-SiNWs are prone to form bundles and rapidly sediment. The sedimentation is accompanied by k-SiNWs flocculation as shown in Fig. 1c. This will impact the homogeneity of the constituents in the electrode, as it will be discussed in the following.

**Figure 2:**
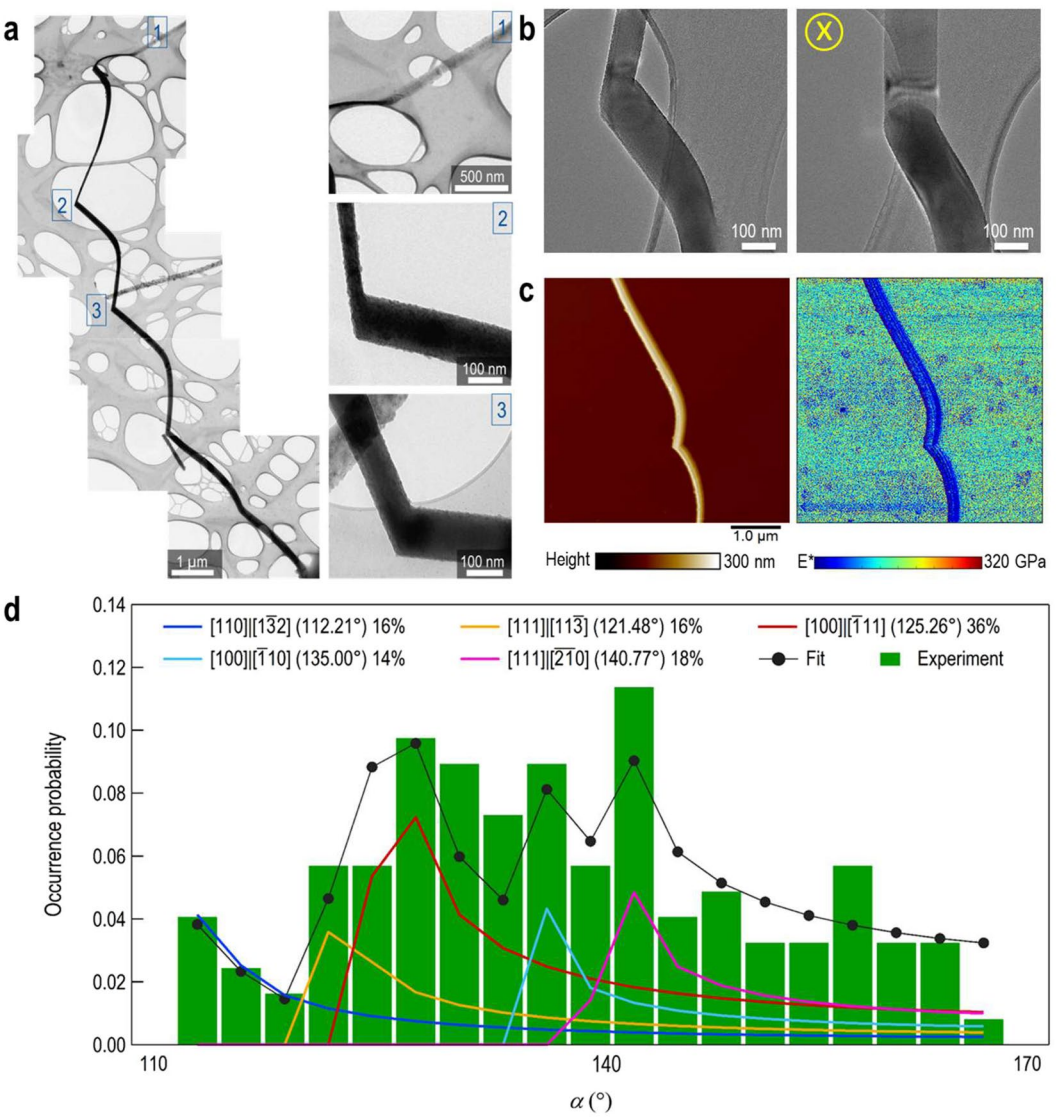
k-SiNWs characterization. TEM image for a k-SiNW obtained by metal assisted chemical etching on a <100> Si substrate, showing the porous segment as well as details on the kinks with different angles between adjacent segments (**a**). Tomography TEM images of the kink revealing the elliptical shape upon tilting of the sample (**b**). Height and effective elastic modulus AFM maps of a SiNW kink with *𝛼* = 120°, confirming that the mechanical stiffness at the kink is similar to the straight segment of the nanowire, as depicted by the color-coded scale bar (**c**). The scan size of images is 5 × 5 µm^2^. Occurrence probability distribution of the measured *𝛼* in SEM along with the least squares fit (**d**). The fitted model gives the distribution of angles formed by pairs of crystallographic directions, as seen from the top. Major contributions are provided by the two most probable pairs of etching directions: <100> - <111> (*𝛼* = 125.26°) and <111> - <210> (*𝛼* = 140.77°).

Figure 2a shows the transmission electron microscopy characterization of a typical k-SiNW, including details on the kinks as well as on the sacrificial porous segment (Figures S1–S6). Upon a detailed microscopic evaluation, we found that these k-SiNWs present a distinctive geometry. First, the diameter of the k-SiNWs before (d_1_) and after (d_2_) the kink considerably differs from the mask’s hole diameter (d_0_). Second, the electron tomography images reveal an elliptical geometry upon tilting of the sample (Fig. 2b and the supplementary videos). This indicates that these k-SiNWs are the result of the downward and radial displacements of the Au mask, triggered by altering the etching kinetics through the reaction interruptions. The nanoscale mechanical properties of individual k-SiNWs were analyzed by intermodulation atomic force microscopy. Figure 2c shows typical height and corresponding effective elastic modulus maps of a kink with *α* ~ 120°. Interestingly, the average effective elastic modulus ~80 GPa does not change along the entire length of the probed segments, and it is close to that of bulk material (see Supplementary information, Material and Methods and Figure S5). This hints at the presence of nanoscale defects on the k-SiNWs surface derived under harsh etching conditions. Overall, the turning angle, *α*, becomes larger as the etching proceeds. In our experiments, *α* is found to take a broad spectrum of values as shown in the occurrence probability distribution from Figs. 2d and S6. Due to the k-SiNWs peculiar morphology, the experimental values of *α* are dependent of the observation angle. Fitting the experimental data with a least squares model helps to identify the most probable crystallographic etching directions (Supplementary Information, Materials and Methods). We found that the most probable pairs of etching directions are: <111> - <100> (*𝛼* = 125.26°) and <111> - <210> (*𝛼* = 140.77°). While there are other reports related to k-SiNWs^34,38^, here we emphasize on the opportunity of multiple crystallographic orientations etching. Experimental findings suggest that the direction of the current etching segment depends on the etching direction of the previous segment. For the first segment, the etching direction is mainly <111> as showed in Figures S1 and S3. The presence of high-viscosity ethanol co-solvent is expected to accelerate the delivery of the reactants and, associated to that, an alteration of Au-Si coupling^39^ that ultimately affects the movement of the Au mask.

## Kinked silicon nanowires-based electrode configuration

**Figure 3:**
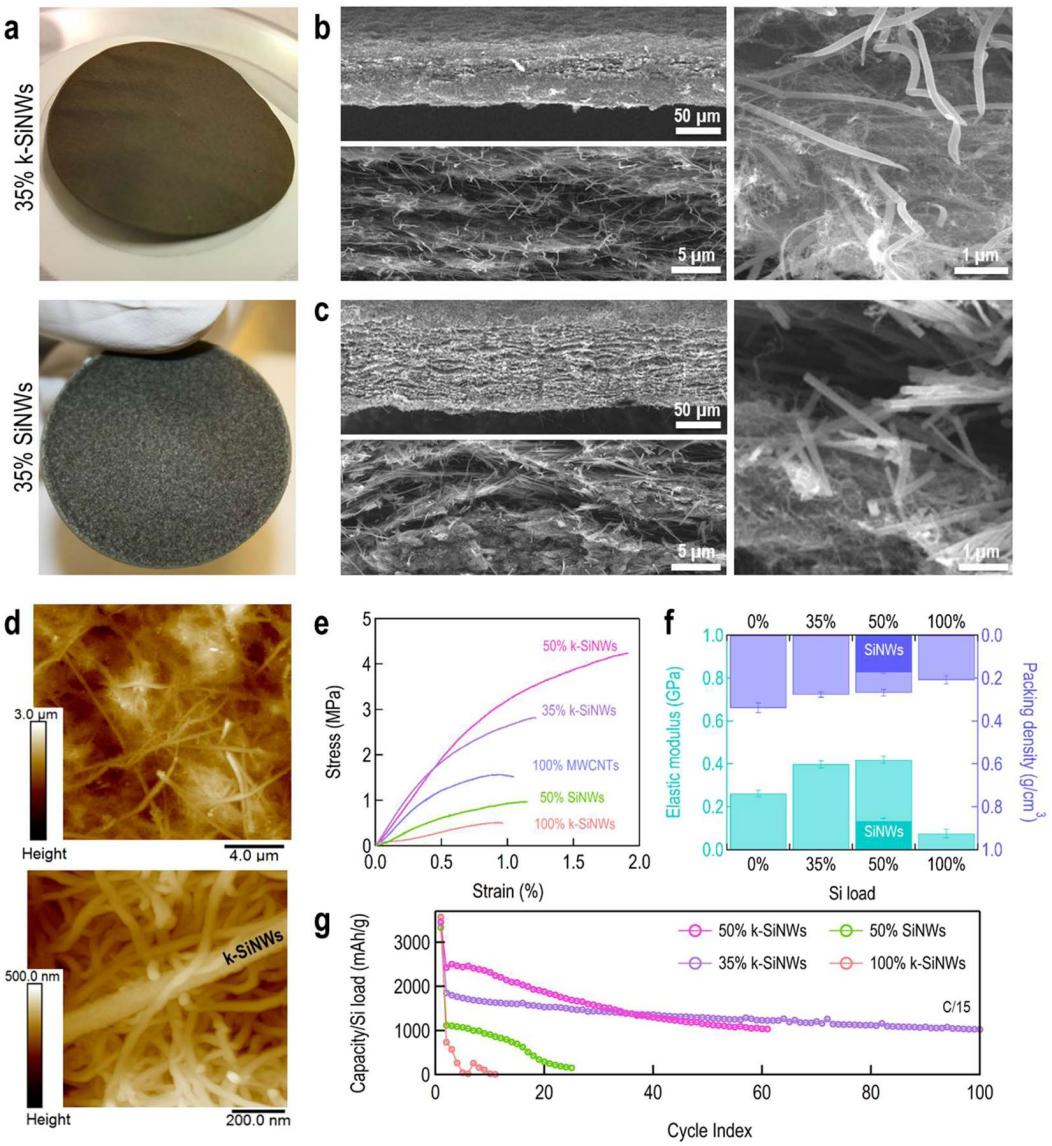
k-SiNWs vs. SiNWs electrode configurations. Optical images of the assemblies with a content of 35% wt. k-SiNWs and 35% wt. SiNWs (**a**). The k-SiNWs assembly presents an uniform color, indicating an homogenous distribution of elements. On the other hand, with the SiNWs-based assembly, distinct regions are identifiable, dominated by either SiNWs or MWCNTs. SEM images with details on the composition of the k-SiNWs assembly (**b**) and SiNWs assembly (**c**). Topography information as revealed by atomic force microscopy (**d**). Characteristic tensile stress-strain curves of the studied assemblies (**e**). Adjustment of elastic modulus (left axis) and packing density (right axis) to the Si load (**f**). Electrochemical performance of the Si-based assemblies evaluated at a rate of C/15 (1 C corresponds to 3600 mA/g) (**g**).

A three-dimensional Si-based interlocking framework is assembled via vacuum-filtration. Self-standing Si-based nanostructured architectures have already been proposed to circumvent the use of common inactive constituents in LIB electrodes like binders, C-based additives or current collectors^40–43^. Eliminating such elements is desirable as it will increase the gravimetric capacity of the electrode. The resulting 100% k-SiNWs assembly is self-standing in contrast to the 100% SiNWs assembly as shown in Figure S7. However, Si’s amophisation is complemented by the loss of the intrinsic electrical conductivity and requires an electroactive additive. In this context, multi-walled carbon nanotubes (MWCNTs) are integrated in the Si-based assemblies. Figure 3a includes optical images of the k-SiNWs and SiNWs-based anode assemblies. By visual inspection, the 35% k-SiNWs-based electrode has a uniform color, indicating the homogenous distribution of the two constituents. On the contrary, the 35% SiNWs-based electrode presents distinct regions dominated by either SiNWs or MWCNTs. The efficient mixing of k-SiNWs with MWCNTs is also confirmed by the cross-section scanning electron microscopy images of the composite from Fig. 3b showing its characteristic interconnected morphology with k-SiNWs randomly distributed in the electrically conducting network of MWCNTs. Such architectures are benefic for LIB anodes since the synergistic interconnectivity creates an interlocking framework, which can accommodate the volume variation during Li insertion, preserving an intimate electrical contact between the k-SiNWs and the MWCNTs (Fig. 3d). This is not expected for SiNWs-based assemblies where a regrouping effect of nanostructures is observed leading to distinct SiNWs or MWCNTs-dominated regions usually separated by voids, and ultimately giving the composite an unstable, layered structure as shown in Figs. 3c and S8a. For a homogenous composite, the available open space is expected to counterbalance the Si volumetric expansion as the lithiation proceeds, resulting in an overall reduced electrode deformation.

In order to explore the advantages of the interweaving k-SiNWs-MWCNTs morphology, mechanical properties are evaluated via tensile tests. Results for macroscopic specimens with 100% MWCNTs, 100% k-SiNWs as well as composites comprising 35%, 50% k-SiNWs and 50% SiNWs are shown in Fig. 3e,f. The tensile strength demonstrated by the 100% k-SiNWs assembly is 0.48 ± 0.01 MPa, while the 100% MWCNTs assembly displays 1.68 ± 0.06 MPa. For the k-SiNWs-based composites, the measured tensile strength is 3.10 ± 0.14 MPa for the 35% k-SiNWs assemblies and 4.00 ± 0.11 MPa for the 50% k-SiNWs assemblies (Figure S8 and Table S1). The 50% SiNWs assemblies are quite fragile exhibiting a tensile strength 1.14 ± 0.08 MPa. Young’s modulus is also found to be dependent of k-SiNWs load. The elastic modulus for 100% MWCNTs is 0.25 ± 0.01 GPa, while for the 35% k-SiNWs-based assembly is 0.39 ± 0.02 GPa. A further increase in the k-SiNWs load to 50% minimally rises the Young’s modulus to 0.41 ± 0.01 GPa, compared to 0.13 ± 0.01 GPa evaluated for the corresponding SiNWs. The enhancement of these mechanical properties, even though the packing density decreases (Fig. 3f), corroborates the three-dimensional interconnectivity provided by the interlocking joints of the k-SiNWs. In the linear regime, mainly MWCNTs reversible stretching is responsible for the observed strain. The presence of kinks reinforces the MWCNTs network and delays the transition to other irreversible degradation mechanisms such as slippage. In addition, these k-SiNWs are expected to be more resilient to stress as they behave like microsprings^4^. For the 100% k-SiWNs assembly, the stretching and slippage of k-SiNWs appear to be concurrent (Figure S8) and responsible for the observed strain hardening. Furthermore, according to these material properties, the k-SiNWs-based assemblies have a foam-like structure. We recall that for an electrode material, porosity is desired as it accommodates volume variations and facilitates electrolyte infiltration. Following this, we evaluated the effect of k-SiNWs loading on the porosity for the studied assemblies (See Supporting Information Table S1 for the physical parameters and the expression used to evaluate porosity). The porosity varies slightly from 0.84 ± 0.01 for the 100% MWCNTs to 0.90 ± 0.01 for the 100% k-SiNWs materials. Intermediate values were found for the 35% k-SiNWs and 50% k-SiNWs composites. A 5% reduction is observed for the 50% k-SiNWs assembly compared to the 50% SiNWs assembly. The spongious nature of the electrode is also confirmed by the force-displacement curves recorded by atomic force microscopy and shown in Figure S9. The available void volume is not enough to accommodate the expected Si swelling, and an overall electrode expansion will occur. This means that the entire electrode will undergo mechanical stresses during cycling, highlighting the importance of mechanically stable electrodes. While there is a certain incentive in lowering the porosity, thus increasing the volumetric capacity, the mechanical integrity of the electrode becomes critical with the Si based anodes. Considering the reported porosity for the traditional slurry-on-metal architecture of Si based anodes^44^, large mechanical stresses are expected to develop during cycling that trigger electrode delamination from the Cu current collector as well as material pulverization, responsible for the limited cycling life. Integrating Si-based materials using graphite-established electrode manufacturing is thus troublesome and clearly points at novel architectures for high capacity Li storage electrodes. The porous k-SiNWs-based self-standing electrodes provide sufficient mechanical strength to sustain the dimensional changes during Si cycling as it will be discussed in the following. We investigated the morphology evolution of the fabricated porous frameworks under deep lithiation conditions and the impact on k-SiNWs volumetric expansion (Figure S10). This was realized by linear sweep voltammetry with the electrode potential held at 10 mV for 10 hours. Under these conditions, the maximum volumetric change is only 25% relative to the pristine state, consistent with other reported values for porous electrodes^45^. The Si extreme volume variation is accommodated by the highly porous assembly that precludes any detrimental rearrangements in the structure of the electrode. Finally, no degradation is observed at the k-SiNWs level.

Next, we evaluated the electrochemical properties of the k-SiNWs-based assemblies with various k-SiNWs content in half-cell configuration with an IL electrolyte system, 1 M lithium bis(trifluoromethanesulfonyl)imide (LiTFSi) in 1-propyl-1-methylpyrrolidinium bis(fluorosulfonyl)imide (PYR_13_FSi), under a constant current mode. For comparison, the 50% SiNWs assembly is also included. A cycling voltammogram of these k-SiNWs electrodes is shown in Figure S11. The obtained capacities as a function of cycle index are displayed in Fig. 3g, while the galvanostatic charge-discharge curves are shown in Figure S12. For the 100% k-SiNWs, the poor performance is attributed to the absence of conductive additive since Si’s intrinsic conductivity is sacrificed in the solid-state amorphisation reaction with Li. On the other hand, the 50% SiNWs electrode suffers from the poor mixing ability of the constituents, isolating regions dominated by the SiNWs from the conductive MWCNTs as the cycling proceeds, visible in the limited cycling life. In terms of cycling stability, it is clear that the 35% k-SiNWs-based assembly stands as the best option while the 50% k-SiNWs electrode demonstrates higher capacities that eventually fade with prolong cycling. Such cycling stability must be related to the homogeneous mechanical properties of the k-SiNWs-based micro-geometry allowing volume variations without affecting the structural integrity of the k-SiNWs-MWCNTs network. Interestingly, while the 50% k-SiNWs electrode also showed improved mechanical properties, the 35% k-SiNWs assembly performs better in terms of cycling performance. This shows that the mechanical properties of the electrode are ultimately a tradeoff of active material load, mixing of the constituents, porosity and the associated packing density. We investigated further the effect of areal loading (k-SiNWs_content_/A_electrode_) on the electrochemical performance, as shown in Figure S13. High areal mass loading is a requisite for adopting Si nanomaterials with the purpose of reducing the weight and the size of the anodes. As the k-SiNWs loading increased from 0.8 to 1.3 mg_Si_/cm^2^, the electrode areal capacity linearly evolved to 2.42 mAh/cm^2^ (1276 mAh/g_electrode_) for the 1.3 mg_Si_/cm^2^. This linear behavior indicates that the two electrode components are active within the studied thickness range (Table S1) and establishes the homogeneous mixing of the constituents. We must emphasize here the benefits of IL cycling compared to the carbonate-based electrolyte system in terms of SEI stability. While in conventional electrolytes, Si’s periodical expansion/contraction enables the continuous formation of SEI, affecting the cycling stability as discussed in Figure S14, for IL systems it was shown that approximately 73% of the Si-based electrode mass gain during the first cycle is preserved throughout cycling^21^. Nevertheless, the underlying degradation mechanism must be related to the charge collection efficiency that is expected to be rather low as the developed SEI isolates both k-SiNWs and MWCNTs, deteriorating the electrical contact, assuming that MWCNTs preserve their electrical properties after extended cycling. Furthermore, on the one hand, studies have revealed that MWCNTs become brittle and prone to sharp fractures after the first lithiation and upon subsequent cycling they break more easily^46^. On the other hand, Si electrodes require passivation for stable cycling.

## Effect of Ni coating on the composite electrochemical properties

**Figure 4:**
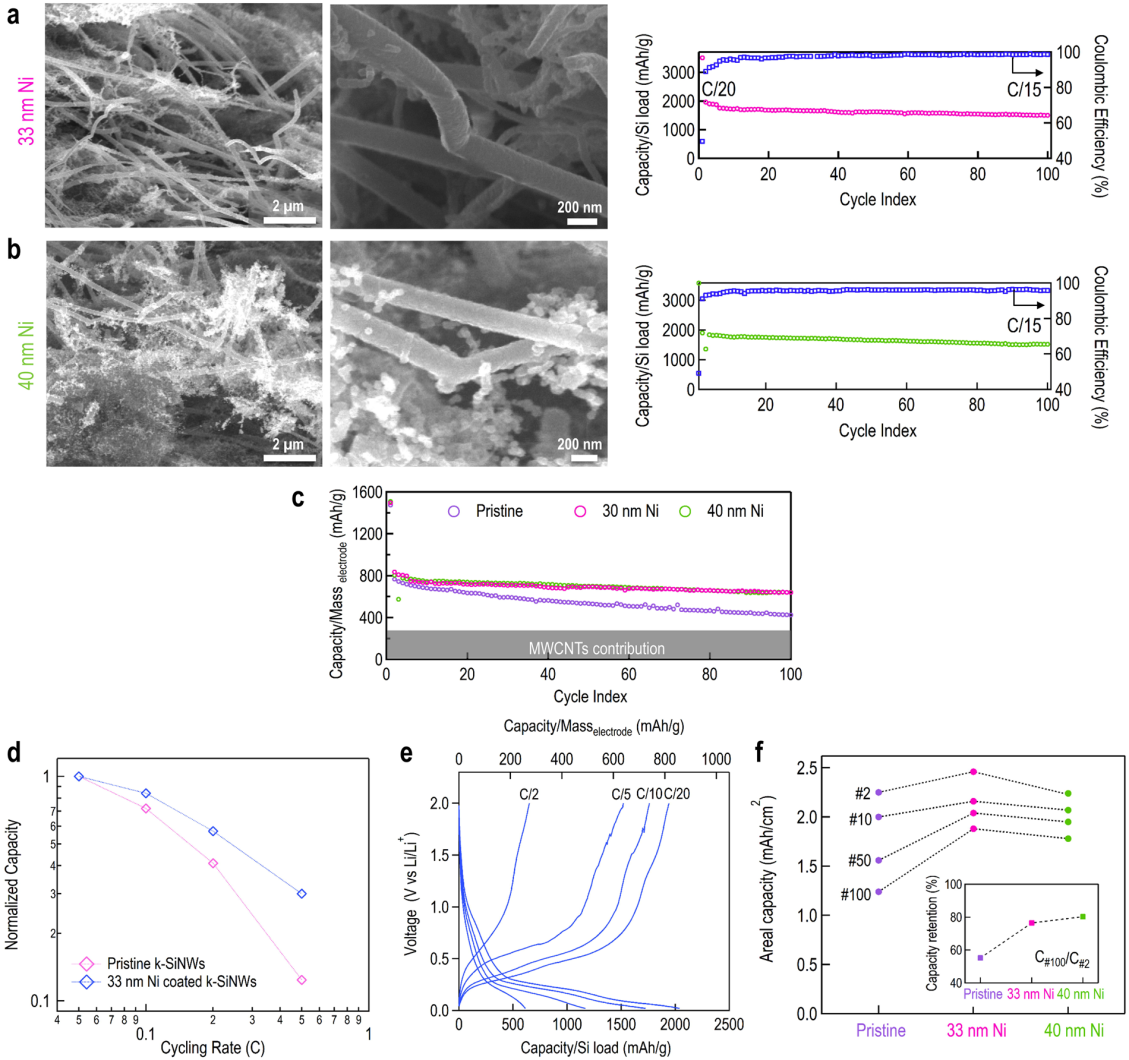
Electrochemical characteristics of 35% wt. k-SiNWs-based electrodes coated with Ni. (**a**) 33 and (**b**) 40 nm Ni-coated assemblies and their associated electrochemical behavior. Capacity (lithiation) normalized to the whole electrode mass (both contributions of Si and MWCNTs) (**c**). After 50 cycles, the capacity for the 40 nm Ni-coated assembly is 700 mAh/g compared to 695 mAh/g for the 33 nm Ni-coated electrode. Normalized capacity as a function of C rate for pristine and 33 nm Ni-coated assemblies with the corresponding galvanostatic charge-discharge profiles (**d**). Charge-discharge profiles according to the capacity per Si load (bottom axis) and the entire electrode load (top axis) (**e**). Areal capacity for the studied electrodes (**f**). At the displayed electrode load (1 mg_Si_/cm^2^), an areal capacity of 2.4 mAh/cm^2^ can be achieved. Regarding the capacity retention, both Ni-coated electrodes present similar characteristics, retaining 80% of the initial capacity (relative to cycle 2) compared to 55% for the pristine assembly.

Next, we selected Ni to investigate the effect of surface passivation on the electrochemical performance of the k-SiNWs-based assembly^18^. In this framework, the anodes containing ~120 nm diameter k-SiNWs are conformally coated with a 33 nm and, alternatively, 40 nm thick Ni layer using an aqueous electroless procedure^47^. Typical SEM images of Ni-coated k-SiNWs systems are shown in Fig. 4, panels a and b. The morphology and the thickness of the Ni shell depend on the plating time: both discontinuous, porous layers and continuous, conformal coatings can be obtained by increasing the deposition time (Figure S15). While a discontinuous, porous Ni layer would mostly improve the electrical conductivity, full structural benefits are expected from continuous, conformal coatings, in particular for thick coatings. Their resulting capacities normalized to the Si content as a function of cycle index is also shown in Fig. 4a,b, while their associated galvanostatic charge-discharge curves are shown in Figure S16. Perhaps of greater importance is the capacity normalized to the entire electrode mass, the Si and MWCNTs content, as shown in Fig. 4c. We observe a capacity of 700 mAh/g_electrode_ for the 33 nm Ni-coated assembly and 695 mAh/g_electrode_ for the 40 nm Ni-coated assembly, compared to 530 mAh/g_electrode_ for the pristine assembly after 50 cycles. This clearly unveils that Si passivation is desirable for stable cycling. The Ni-coated electrodes also present an areal capacity close to 2 mAh/cm^2^ after 50 cycles, a value required for commercial usage, as shown in Fig. 4f. This behavior is primary attributed to benefits stemming from the Ni coating, mainly the mechanical shielding and the electrical characteristics of the shell. Adopting such core-shell systems can have a powerful impact on the battery sizing, since similar capacities can be obtained using less than half of the active material packed in half of the thickness of the conventional graphite electrode. Regarding the cycling stability, after 100 cycles, the capacity retention for the 33 and 40 nm Ni-coated electrodes is 76.5 and 80.3%, respectively, compared to 55.2% for the pristine electrode. Considering the similar electrochemical performances, using the 40 nm Ni-coated k-SiNWs is not justified, since whenever possible, inactive materials should be eliminated. As previously discussed, another profit of the Ni coating is to provide efficient charge collection, which is expected to have a positive impact on the electrochemical rate performance. The rate capability plots (Figs. 4 and S17) highlight the amplitude of the specific capacity at different C-rates for the as-prepared and 33 nm Ni-coated k-SiNWs anode assemblies. The magnitude of the Li uptake of the 33 nm Ni-coated k-SiNWs anode assembly is considerably greater than the as-prepared one, in particular at high rates. This response is evidence of favorable electronic pathways in the 33 nm Ni-coated k-SiNWs electrodes. Nevertheless, the capacity reduces as the current density increases. This behavior is explained by the Si sluggish lithiation concurrent with limited lithium-ion conductivity of the ionic liquid electrolyte at room temperature. The same benefits are observed for discontinuous Ni-coated k-SiNWs assemblies, as shown in Figures S18 and S19, confirming the importance of good charge collection in co-integrated anodes and current collectors.

**Figure 5:**
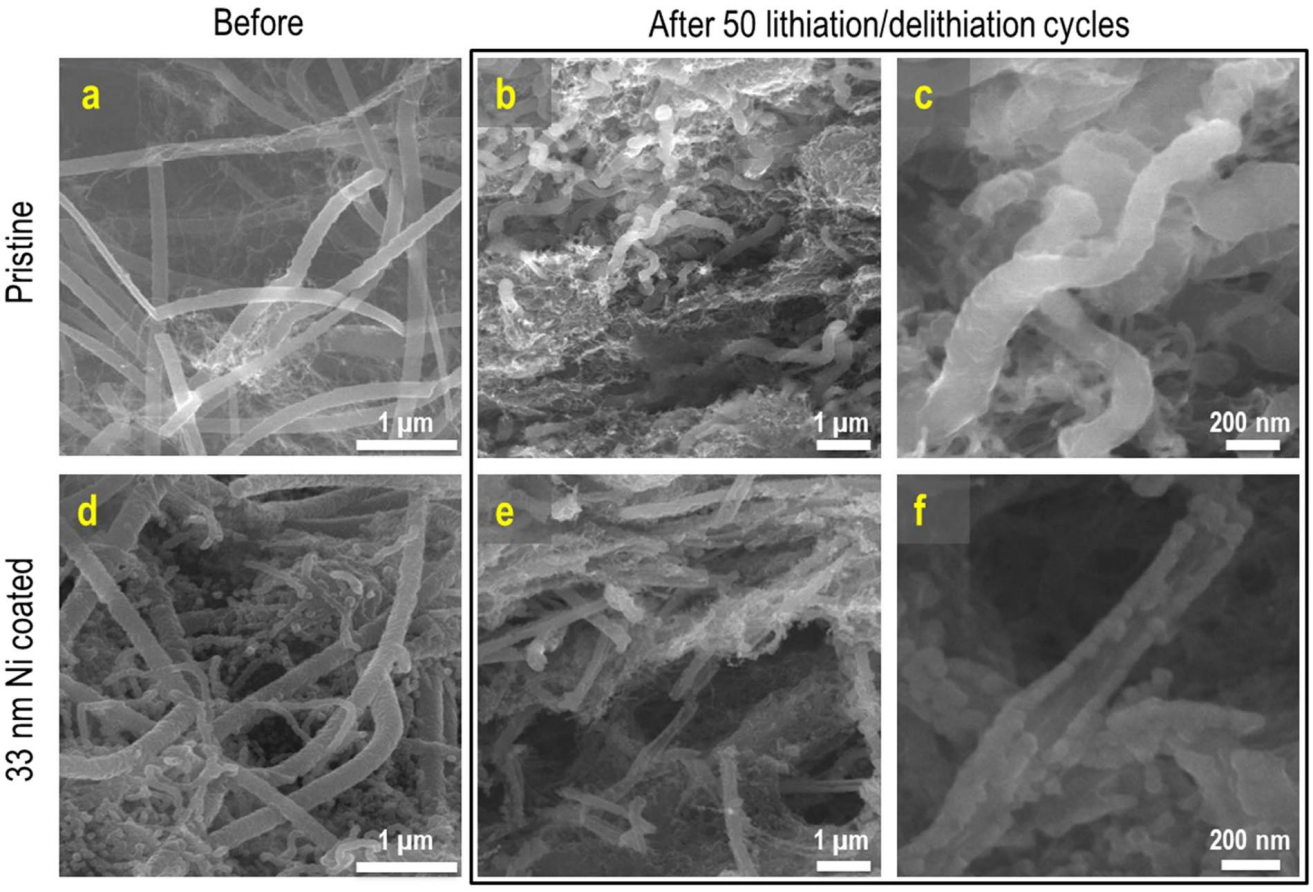
Post mortem analysis of the electrodes upon charge-discharge cycles. SEM images of pristine (**a**) and Ni-coated k-SiNWs assemblies (**d**). Morphology change after 50 lithiation/delithiation cycles: pristine (**b**,**c**) and Ni-coated (**e**,**f**) k-SiNWs assemblies. The Ni coating cracks upon Li insertion but the resulting configuration remains attractive, as it resembles the Si thin-film behavior. Si maintains an intimate contact with the electronically active Ni coating resulting in better charge collection efficiency, while being partially isolated from the electrolyte.

The post mortem analysis of the electrodes after 50 lithiation/delithiation cycles is presented in Fig. 5. The structural integrity of the as-prepared electrode appears to be preserved upon cycling. Indeed, for small diameters, under deep lithiation conditions, the swelling anisotropy of SiNWs is less pronounced^18,48^. The same observation is valid for k-SiNWs: they sustain the Li uptake by preserving the turning angle with no particular deformations (Figure S10). Nevertheless, after intense cycling, the kinks become indistinguishable as the k-SiNWs progressively become smoother and curved. Although elucidating the mechanism responsible for this behavior requires further investigation, an important contribution can be assigned to the unique interface between two different orientation segments and their corresponding swelling anisotropy as given by the crystallographic-depended reaction kinetics. The Ni coating uniformly unzips, presenting a long, longitudinal opening, typically offering the Ni-coated k-SiNWs a single fracture site as highlighted in Fig. 5e,f. Overall, after 50 cycles, the Ni-coated k-SiNWs maintain their shape determined by the first lithiation step, due to the structural stiffness induced by the Ni shell. This correlates with the observed high cycling stability of the 33 nm Ni-coated k-SiNWs anode assembly compared to the pristine assembly. Despite the cracks, the electrical conductivity is preserved and plays a fundamental role in the prevention of the capacity decay. Nonetheless, even with this electrode configuration, Si pulverization is leading to capacity degradation. While we explored the feasibility of k-SiNWs as active material for lithium ion battery electrodes and used Ni as a model system to further emphasize passivation as a requirement for stable cycling of Si-based anodes, the 33 nm-thick conformal coating of k-SiNWs is expected to increase by 20% the weight of the integrated current collector compared to the conventional Cu foil. In this sense, lighter functional materials such as conducting polymers can successfully replace the metallic current collector and enable full exploitation of Si anodes^49,50^.

In summary, a continuous metal assisted chemical etching sustained by chemical peeling method is proposed for the controlled fabrication of k-SiNWs. This procedure is versatile and enables the fabrication of customized k-SiNWs. A chemical peeling step is introduced to facilitate the ultrasonic collection of k-SiNWs and allows for the reuse of the Si substrate. We have investigated the benefits of such nanostructures as LIB electrodes and proposed a self-standing electrode with k-SiNWs and MWCNTs as building blocks. We challenged the current slurry-on-metal anode paradigm by introducing a highly porous, interweaving k-SiNWs-based electrode with minimal deformation during cycling and an integrated Ni current collector for effective Si passivation and efficient electrical pathways. Such architectures seem more suitable than the conventional slurry-on-metal design to sustain long cycling of Si-based anodes. The presence of kinks positively influences the electrode assembly by: (i) facilitating a homogenous distribution of constituents, (ii) reinforcing the electrode framework to allow further processing, and (iii) delaying the electrode degradation during structural changes associated with Li shuttling. The Si passivation with Ni locks the electrode framework and prevents component reorganization during cycling. The enhanced electrochemical performance of the electrodes results from the synergy between the k-SiNWs three-dimensional interlocking features and the critical contributions of the Ni coating associated with the robust SEI film activated by the ionic liquid electrolytes.

## Electronic supplementary material

Supplementary video 1

Supplementary Video 2

Supplementary Information

## References

[1] Armand M, Tarascon J-M (2008). Building better batteries. Nature.

[2] Goodenough JB, Kim Y (2010). Challenges for Rechargeable Li Batteries. Chem. Mater..

[3] Molina Piper D (2016). Optimized Silicon Electrode Architecture, Interface, and Microgeometry for Next-Generation Lithium-Ion Batteries. Adv. Mater..

[4] Polat BD, Keles O, Amine K (2015). Silicon-Copper Helical Arrays for New Generation Lithium Ion Batteries. Nano Lett..

[5] Kovalenko I (2011). A Major Constituent of Brown Algae for Use in High-Capacity Li-Ion Batteries. Science.

[6] Chan CK (2008). High-performance lithium battery anodes using silicon nanowires. Nat. Nanotechnol..

[7] Beaulieu LY, Eberman KW, Turner RL, Krause LJ, Dahn JR (2001). Colossal Reversible Volume Changes in Lithium Alloys. Electrochem. Solid-State Lett..

[8] Pharr M, Zhao K, Wang X, Suo Z, Vlassak JJ (2012). Kinetics of Initial Lithiation of Crystalline Silicon Electrodes of Lithium-Ion Batteries. Nano Lett..

[9] Liu N (2014). A pomegranate-inspired nanoscale design for large-volume-change lithium battery anodes. Nat. Nanotechnol..

[10] Magasinski A (2010). High-performance lithium-ion anodes using a hierarchical bottom-up approach. Nat. Mater..

[11] Jing S, Jiang H, Hu Y, Shen J, Li C (2015). Face-to-Face Contact and Open-Void Coinvolved Si/C Nanohybrids Lithium-Ion Battery Anodes with Extremely Long Cycle Life. Adv. Funct. Mater..

[12] Gao B, Sinha S, Fleming L, Zhou O (2001). Alloy Formation in Nanostructured Silicon. Adv. Mater..

[13] Nadimpalli SPV (2012). Quantifying capacity loss due to solid-electrolyte-interphase layer formation on silicon negative electrodes in lithium-ion batteries. J. Power Sources.

[14] Karki K (2013). Hoop-Strong Nanotubes for Battery Electrodes. ACS Nano.

[15] Wu H (2012). Stable cycling of double-walled silicon nanotube battery anodes through solid-electrolyte interphase control. Nat. Nanotechnol..

[16] Lee S (2014). Surface-Coverage-Dependent Cycle Stability of Core-Shell Nanostructured Electrodes for Use in Lithium IonBatteries. Adv. Energy Mater..

[17] Cui L-F, Ruffo R, Chan CK, Peng H, Cui Y (2009). Crystalline-Amorphous Core−Shell Silicon Nanowires for High Capacity and High Current Battery Electrodes. Nano Lett..

[18] Sandu G (2014). Surface Coating Mediated Swelling and Fracture of Silicon Nanowires during Lithiation. ACS Nano.

[19] Dalavi S, Guduru P, Lucht BL (2012). Performance Enhancing Electrolyte Additives for Lithium Ion Batteries with Silicon Anodes. J. Electrochem. Soc..

[20] Chockla AM (2012). Influences of Gold, Binder and Electrolyte on Silicon Nanowire Performance in Li-Ion Batteries. J. Phys. Chem. C.

[21] Piper DM (2015). Stable silicon-ionic liquid interface for next-generation lithium-ion batteries. Nat. Commun..

[22] Huang Z, Geyer N, Werner P, de Boor J, Gösele U (2011). Metal-Assisted Chemical Etching of Silicon: A Review. Adv. Mater..

[23] Huang Z (2008). Extended Arrays of Vertically Aligned Sub-10 nm Diameter [100] Si Nanowires by Metal-Assisted Chemical Etching. Nano Lett..

[24] Plass KE (2009). Flexible Polymer-Embedded Si Wire Arrays. Adv. Mater..

[25] Weisse JM, Lee CH, Kim DR, Zheng X (2012). Fabrication of Flexible and Vertical Silicon Nanowire Electronics. Nano Lett..

[26] Weisse JM (2013). Electroassisted Transfer of Vertical Silicon Wire Arrays Using a Sacrificial Porous Silicon Layer. Nano Lett..

[27] Huang Z, Fang H, Zhu J (2007). Fabrication of Silicon Nanowire Arrays with Controlled Diameter, Length, and Density. Adv. Mater..

[28] Peng K, Lu A, Zhang R, Lee S-T (2008). Motility of Metal Nanoparticles in Silicon and Induced Anisotropic Silicon Etching. Adv. Funct. Mater..

[29] Chen C-Y, Wu C-S, Chou C-J, Yen T-J (2008). Morphological Control of Single-Crystalline Silicon Nanowire Arrays near Room Temperature. Adv. Mater..

[30] Huang Z (2010). Oxidation Rate Effect on the Direction of Metal-Assisted Chemical and Electrochemical Etching of Silicon. J. Phys. Chem. C.

[31] Kim J (2011). Au/Ag Bilayered Metal Mesh as a Si Etching Catalyst for Controlled Fabrication of Si Nanowires. ACS Nano.

[32] Huang Z (2009). Ordered Arrays of Vertically Aligned [110] Silicon Nanowires by Suppressing the Crystallographically Preferred <100> Etching Directions. Nano Lett..

[33] Geyer N (2012). Model for the Mass Transport during Metal-Assisted Chemical Etching with Contiguous Metal Films As Catalysts. J. Phys. Chem. C.

[34] Kim J, Kim YH, Choi S-H, Lee W (2011). Curved Silicon Nanowires with Ribbon-like Cross Sections by Metal-Assisted Chemical Etching. ACS Nano.

[35] Bai, F., To, W.-K. & Huang, Z. Porosification-Induced Back-Bond Weakening in Chemical Etching of n-Si(111). *J. Phys. Chem*. C **117**, 2203–2209 (2013).

[36] Lai CQ, Choi WK (2014). Synthesis of free-standing, curved Si nanowires through mechanical failure of a catalyst during metal assisted chemical etching. Phys. Chem. Chem. Phys..

[37] Chiappini C, Liu X, Fakhoury JR, Ferrari M (2010). Biodegradable Porous Silicon Barcode Nanowires with Defined Geometry. Adv. Funct. Mater..

[38] Chen Y (2017). Controlling Kink Geometry in Nanowires Fabricated by Alternating Metal-Assisted Chemical Etching. Nano Lett..

[39] Lai CQ, Cheng H, Choi WK, Thompson CV (2013). Mechanics of Catalyst Motion during Metal Assisted Chemical Etching of Silicon. J. Phys. Chem. C.

[40] Hwang C, Kim T-H, Cho Y-G, Kim J, Song H-K (2015). All-in-one assembly based on 3D-intertangled and cross-jointed architectures of Si/Cu 1D-nanowires for lithium ion batteries. Sci. Rep..

[41] Hu L, Wu H, La Mantia F, Yang Y, Cui Y (2010). Thin, Flexible Secondary Li-Ion Paper Batteries. ACS Nano.

[42] Cui L-F, Hu L, Choi JW, Cui Y (2010). Light-Weight Free-Standing Carbon Nanotube-Silicon Films for Anodes of Lithium Ion Batteries. ACS Nano.

[43] Evanoff K (2012). Ultra Strong Silicon-Coated Carbon Nanotube Nonwoven Fabric as a Multifunctional Lithium-Ion Battery Anode. ACS Nano.

[44] Higgins TM (2016). A Commercial Conducting Polymer as Both Binder and Conductive Additive for Silicon Nanoparticle-Based Lithium-Ion Battery Negative Electrodes. ACS Nano.

[45] Piper DM (2014). Hierarchical Porous Framework of Si-Based Electrodes for Minimal Volumetric Expansion. Adv. Mater..

[46] Liu Y (2011). Lithiation-Induced Embrittlement of Multiwalled Carbon Nanotubes. ACS Nano.

[47] Lancaster JR (2008). Toward a Universal Method To Pattern Metals on a Polymer. Chem. Mater..

[48] Lee SW, McDowell MT, Berla LA, Nix WD, Cui Y (2012). Fracture of crystalline silicon nanopillars during electrochemical lithium insertion. Proc. Natl. Acad. Sci..

[49] Sandu G (2017). Mechanochemical Synthesis of PEDOT:PSS Hydrogels for Aqueous Formulation of Li-Ion Battery Electrodes. ACS Appl. Mater. Interfaces.

[50] Choi S, Kwon T, Coskun A, Choi JW (2017). Highly elastic binders integrating polyrotaxanes for silicon microparticle anodes in lithium ion batteries. Science.

