# Supplementary material for: Kinked silicon nanowires-enabled interweaving electrode configuration for lithium-ion batteries
Source: Sci Rep. 2018 Jun 28;8:9794. doi: 10.1038/s41598-018-28108-3 (PMC6023865; doi:10.1038/s41598-018-28108-3)
Supplement: Supplementary file 3 — Supplementary Information [file 41598_2018_28108_MOESM3_ESM.pdf]

Supporting Information for

Kinked silicon nanowires-enabled interweaving electrode configuration for lithium-ion batteries

Georgiana Sandu[1], Michael Coulombier[2], Vishank Kumar[3], Hailu G. Kassa[4], Ionel Avram[1], Ran Ye[1], Antoine Stopin[5,6], Davide Bonifazi[5], Jean-François Gohy[3], Philippe Leclère[4], Xavier Gonze[3], Thomas Pardoen[2], Alexandru Vlad[3], Sorin Melinte[1]*

[1]Institute of Information and Communication Technologies, Electronics and Applied Mathematics, Université catholique de Louvain, 1348 Louvain-la-Neuve, Belgium

[2]Institute of Mechanics, Materials, and Civil Engineering, Université catholique de Louvain, 1348 Louvain-la-Neuve, Belgium

[3]Institute of Condensed Matter and Nanosciences, Université catholique de Louvain, 1348 Louvain-la-Neuve, Belgium

[4]Laboratory for Chemistry of Novel Materials, Center for Innovation and Research in Materials and Polymers, University of Mons, 7000 Mons, Belgium

[5]School of Chemistry, Cardiff University, Park Place, Main Building, Cardiff CF10 3AT, United Kingdom

[6]Department of Chemistry, University of Namur, Rue de Bruxelles 61, 5000 Namur, Belgium

[*]Corresponding author: sorin.melinte@uclouvain.be

Contents

Materials and methods. Experimental details. Detailed descriptions of the continuous fabrication of kinked silicon nanowires enabled by the chemical peeling step, TEM and SEM investigations of kinks, the mathematical formalism of the fitting model used to comprehend the broad spectrum of angles observed for the kinks, a detailed comparison between vertical SiNWs and k-SiNWs, a listing of the mechanical properties for the evaluated assemblies, detailed electrochemical analysis, Ni electroless deposition, morphology evolution of the k-SiNWs electrodes after lithiation and lithiation/delithiation by cyclic voltammetry.

Supplementary video SV1. Representative TEM movie of a kinked Si nanowire over a tilt range of ±65° with tilt increments of 5°. Frame rate is 10 images/s. Field of view is 400 x 400 nm. Total length is 2.56 s (AVI).

Supplementary video SV2. Representative movie of the 3D reconstruction of a kinked Si nanowire. Frame rate is 15 images/s. Total length is 13.33 s (AVI).

Materials and methods

Kinked silicon nanowires (k-SiNWs) synthesis.

Colloidal lithography: Silicon substrates (*p*-type, <100>, resistivity 10-25 Ωcm) have been used after 10 minutes ultrasonic treatment in methanol. Next, a short oxygen plasma treatment was performed to render their surface hydrophilic for the colloidal processing. A monolayer of 260 nm nominal diameter polystyrene (PS) spheres (Microparticles Gmbh) was assembled on the pre-treated Si substrate. In the next step, reactive ion etching was used to reduce the PS sphere diameter to 120 nm, while retaining the initial close-packed hexagonal ordering. A 15 nm Au layer is subsequently deposited on the PS-decorated Si substrate by physical vapor deposition. Adhesive tape followed by an overnight stay in dichloromethane was employed for the lift-off of the PS spheres.

Metal assisted chemical etching (MACE): The MACE was performed in dark, ambient conditions with no agitation using ethanol as co-solvent. The etching of k-SiNWs required two consecutive steps. First, the Au-patterned Si substrate was etched in a solution comprising 9.3M HF and 1.6M H_2O_2 (ε = $[HF]/[H_2O_2]$ = 5.8) for 5 minutes. The reaction is quenched in methanol for less than 1 minute.

The second etching step employed for the k-SiNWs fabrication relied on a series of etch-quench sequences with 5 minutes etching in an etchant with 14M HF and 1.3M H_2O_2 followed by a 1 minute quench in methanol. The complete MACE process lasts a total time of 41 minutes. The k-SiNWs formed during this time typically had 6 kinks and 40 µm in length.

Chemical peeling: The sequential, top-down etching approach used in the fabrication of the k-SiNWs allows for the creation of a final porous nanowire segment. For this purpose, the etching was performed in a solution containing 4.6M HF and 8.3M H_2O_2 for 3 minutes (Figure S1). The k-SiNWs were subsequently collected in methanol with a 10 seconds ultrasonic treatment.

k-SiNWs-based assembly with multi-walled carbon nanotubes (MWCNTs). The collected k-SiNWs are mixed with pre-dispersed MWCNTs (Nanocyl 7000) in methanol. The solution was further filtered through a Millipore system using a 0.45 µm nylon filter. The resulting composite was left to dry overnight in a vacuum oven at 50°C.

Electroless Ni deposition. The electroless deposition procedure was adopted from literature.[1] Briefly, the composite is immersed in an aqueous plating bath containing 1 g of nickel sulfate hexahydrate (Sigma Aldrich) as the source of Ni ions, 0.05 g dimethylamine borane (Acros Organic) as reducing agent, 0.5 g of sodium citrate (Sigma Aldrich) and 0.25 g of lactic acid (Sigma Aldrich) as complexant and, respectively, buffer in 50 ml deionized water. The pH was adjusted to 7 with ammonium

hydroxide. The plating was performed at room temperature under continuous agitation. The onset of deposition was marked by the emergence of gas bubbles. After this point, the immersion time varied from 10 to 25 minutes depending on the desired thickness (Figure S15). After the plating, the assembly was intensively rinsed in deionized water and left to dry in air. Morphology studies of the Ni coatings were mainly carried by scanning electron microscopy (SEM).

Electrochemical measurements. For the pristine assemblies, 500 nm of Cu was deposited on one side of the assembly as current collector by physical vapor deposition.

The electrochemical performances were recorded using a battery analyzer (Arbin Instruments). Coin-cells were assembled with either pristine k-SiNWs-based assembly or Ni-coated k-SiNWs-based assembly as the working electrode and Li metal foils as counter and reference electrodes. The electrolyte systems used were 1M LiTFSi in $PYR_{13}FSi$ (Solvionic) and 1M $LiPF_6$ in EC:DEC (2% VC) (Solvionic). The separator was a Celgrad membrane for the organic electrolyte and a glass micro-fiber disk (Whatman GFD) for the ionic liquid electrolyte. The galvanostatic charge-discharge curves were recorded between 0.02 to 2 V vs Li/Li^+. The half-cells were discharged (lithiated) and charged (delithiated) at different current densities as indicated throughout the main text.

Atomic force microscopy (AFM) analysis. The morphology of the assemblies of Si nanowires and carbon nanotubes was inspected by a Dimension Icon AFM (Bruker) in tapping mode using silicon cantilevers (Bruker) with a force constant of ~42 N/m and an apex with a radius of curvature ~10 nm.

Intermodulation atomic force microscopy (ImAFM). ImAFM uses an external multi-frequency lock-in amplifier to excite the cantilever at two frequencies close to the resonance frequency peak and then it records the photo-detector response at many intermodulation products of the two drives.[2] Like any other AFM technique, the surface scanning is normally controlled by the host AFM. Essentially, the ImAFM measures the amplitude dependence of the tip-surface force at each pixel of an image while scanning; thus, both amplitude and phase images are collected.[3,4]

ImAFM uses a built-in method to determine the tip-surface force from the measured tip motion. Normally, this procedure requires certain assumptions for the tip-surface force to reconstruct the measured data. Specifically, it reconstructs the tip-surface force $F(d)$ as a function of cantilever deflection $d = z - h$, where h (the equilibrium tip height) and z (the tip position) are measured from a fixed position in the reference frame where the sample is at rest. Following Platz *et al.,* we have approximated the nonlinear tip–surface force by a polynomial of finite degree N in the cantilever

deflection as $F(d) = \sum_{j=1}^{N} g_i\, d^j$.[5] The polynomial coefficients (in our case N = 19) g_j can be obtained from the measured spectrum of odd-order intermodulation products. Once the polynomial approximation is selected to reconstruct the force curves as a function of cantilever deflection, we can use a specific force model to fit the data. For our samples, k-SiNWs deposited on oxidized Si(100) substrates, we have selected the Derjaguin - Muller - Toporov (DMT) model[6] considering a spherical tip and a flat surface to fit the following polynomial function:

$$F(z) = \begin{cases} -F_{min}\dfrac{a_0^2}{(z+h+a_0)^2} & ,z > -h \\ -F_{min} + \dfrac{4}{3}E^*\sqrt{R(-z-h)^3} & ,z \leq -h\,. \end{cases}$$

Here, R is the tip radius, a_o is the interatomic distance, F_{min} is the depth of the force minimum, and E^* represents the effective elastic modulus.

Transmission electron microscopy (TEM) analysis. TEM analysis and electron tomography were performed on a Jeol JEM2100 transmission electron microscope microscope operated at 200 kV. TEM images for electron tomography were collected over a tilt range of ±65° with tilt increments of 5°, at a nominal magnification of 35000 – 60000 using the SerialEM software. The 3D reconstructions of the aligned tilt series were obtained using the simultaneous iterative reconstruction technique. Image filtering and alignment was done using Tomviz and the reconstruction performed using BrightfieldGui. Final image rendering was then done in Tomviz.

Least squares fitting of the measured *α* distribution. To understand the observed broad distribution of α, we use the least squares method to fit the recorded experimental data.

Model. Let's consider a kinked nanowire AB with the kink at the location K as shown in Scheme 1. The angle formed by the arms AK and BK is θ, such that when θ = 180°, the nanowire is straight and when θ = 0°, the nanowire folds backward.

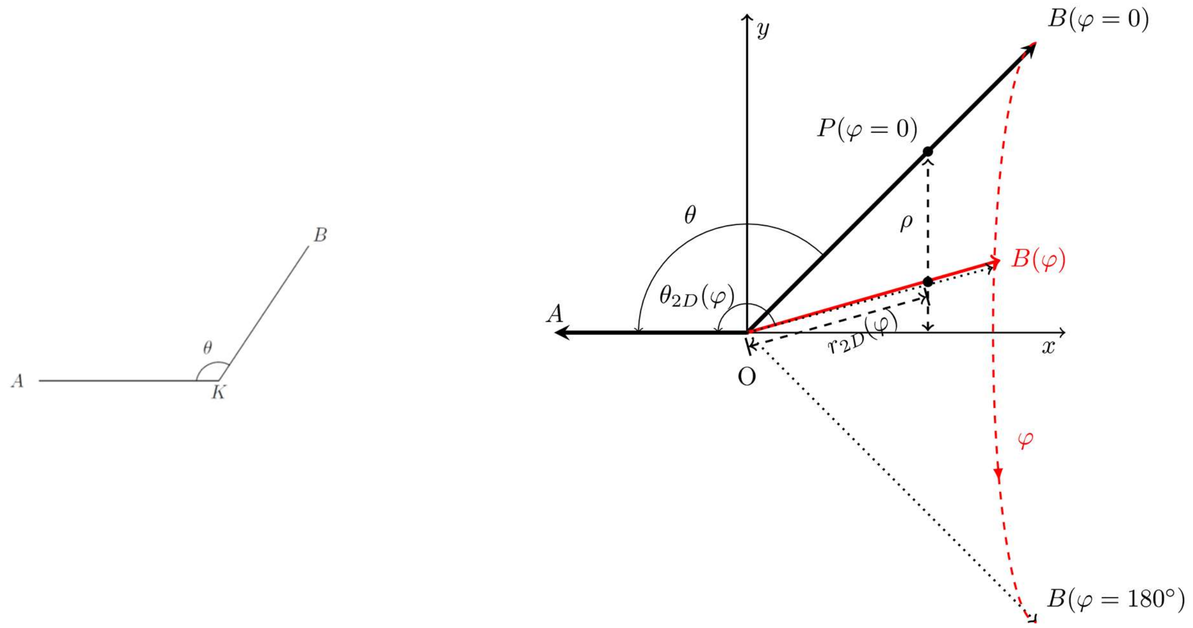

Scheme 1: (Left panel) A kinked nanowire *AB*. (Right panel) The nanowire segment *AK* is positioned along cartesian axis *x*, while the viewing direction is in the *z* direction and varying as a function of angle φ with respect to the *x* axis. Any marking in the *z* direction is shown in red and the remaining markings in the *xy* plane are illustrated in black.

The kink is viewed in a direction that might not be perpendicular to the plane in which the whole nanowire is situated. Therefore, the observed angle α will be the projection (θ_{2D}) of the angle θ that depends on the alignment of viewing plane with respect to the plane of the nanowire (and it varies from θ to $2\pi - \theta$). In this model, we have assumed that the viewing plane is parallel to the axis AK as shown in Scheme 1 with the following conditions:

- Arm AK is in the x direction;
- Viewing direction is the *z* direction;
- The arm BK is rotated, while keeping AK unchanged, and the viewing direction unchanged;
- Point K is at the origin O.

The angle φ denotes the rotation of the arm BK around the x axis, and is set to 0 when the arm BK is in the xy-plane. The coordinates of a point *P* on the arm BK will be:

$$\vec{r}(\varphi)(x, y(\varphi), z(\varphi)) = (x, \rho\cos\varphi, \rho\sin\varphi). \quad (1)$$

The projected angle $\theta_{2D}(\varphi)$ will be such that:

$$x(\varphi) = -r_{2D}(\varphi)\cos\theta_{2D}(\varphi), \quad (2)$$

$$y(\varphi) = r_{2D}(\varphi)\sin\theta_{2D}(\varphi), \quad (3)$$

where $r_{2d}(\varphi)$ is the viewed length of the segment OP.

Thus, $\rho\cos\varphi = -\tan\theta_{2D}(\varphi) \cdot x$. (4)

Moreover, at $\varphi = 0, \rho = -tan\theta \cdot x$, (5)

$$cos\varphi \cdot tan\theta = \tan\theta_{2D}(\varphi). \quad (6)$$

This Equation 6 relates the apparent angle θ_{2D} (*i.e.* α) with the real kink angle θ, and the rotation angle φ.

Distribution of viewing angles. Assuming that the distribution of φ angles is homogeneous, from 0° to 180° (0 to π), meaning that the nanowire do not penetrate the $z < 0$ region, the distribution function is $g(\varphi)$ and it integrates to 1 between 0 and $\boldsymbol{\pi}$.

$$\int_0^{\pi} g(\varphi)d\varphi = 1 \Rightarrow g(\varphi) = \frac{1}{\pi}. \quad (7)$$

We must find $\tilde{g}(\theta_{2D})$ corresponding to $g(\varphi)$ with the normalization condition:

$$\int_{\theta_{2D}(0)}^{\theta_{2D}(\pi)} \tilde{g}(\theta_{2D})d\theta_{2D} = 1. \quad (8)$$

From Equation 6, we can write:

$$\cos\varphi = \frac{\tan\theta_{2D}(\varphi)}{\tan\theta}. \quad (9)$$

Differentiating both sides,

$$-\sin\varphi \cdot d\varphi = \frac{d\theta_{2D}}{tan\theta \cdot cos^2\theta_{2D}(\varphi)},$$

$$\frac{1}{\pi} \cdot d\varphi = -\frac{1}{\pi} \cdot \frac{1}{sin\varphi \cdot tan\theta \cdot \cos^2\theta_{2D}(\varphi)} \cdot d\theta_{2D.} \quad (10)$$

Moreover, when $\varphi = 0$, $\theta_{2D}(0) = \theta$, and when $\varphi = \pi$, $\theta_{2D}(\pi) = 2\pi - \theta$ [if $\theta > \pi/2$], $\theta_{2D}(\pi) = -\theta$ [if $\theta < \pi/2$].

Thus, if $\theta > \pi/2$, $\tilde{g}(\theta_{2D}) = -\frac{1}{\pi \cdot \sin\varphi \cdot \tan\theta \cdot \cos^2\theta_{2D}}$; $\theta_{2D} \in (\theta, 2\pi - \theta)$. (11)

If $\theta < \pi/2$, also $\tilde{g}(\theta_{2D}) = -\frac{1}{\pi \cdot \sin\varphi \cdot \tan\theta \cdot \cos^2\theta_{2D}}$; $\theta_{2D} \in (\theta, -\theta)$. (12)

For $\theta > \pi/2$, substituting $\sin\varphi$ from Equation 9 as $\sin\varphi = \sqrt{1 - \frac{\tan^2\theta_{2D}}{\tan^2\theta}}$, we obtain

$$\tilde{g}(\theta_{2D}) = \frac{1}{\pi \cdot \sqrt{\tan^2\theta - \tan^2\theta_{2D}} \cdot \cos^2\theta_{2D}}; \theta_{2D} \in (\theta, 2\pi - \theta). \quad (13)$$

Using the rotational symmetry of angle φ from 0 to $\pi/2$ and $\pi/2$ to π, the distribution function for φ between 0 and $\pi/2$ can be written as:

$$\text{if } \theta > \pi/2, \quad \tilde{g}(\theta_{2D}) = \frac{2/\pi}{\sqrt{\tan^2\theta - \tan^2\theta_{2D}} \cdot \cos^2\theta_{2D}}; \theta_{2D} \in (\theta, \pi), \quad (14)$$

$$\text{if } \theta < \pi/2, \quad \tilde{g}(\theta_{2D}) = \frac{2/\pi}{\sqrt{\tan^2\theta - \tan^2\theta_{2D}} \cdot \cos^2\theta_{2D}}; \theta_{2D} \in (0, \theta). \quad (15)$$

Assuming the overall distribution function is a linear sum of all the individual distributions associated with θ_i, the expected distribution function can be written as:

$$g_{exp}(\theta_{2D}) = \sum_i \tilde{g}_{\theta_i}(\theta_{2D}) \cdot w_i, \quad (16)$$

where w_i are the weights assigned to each θ_i.

Least squares fit. The angles formed between pairs of low indices crystallographic directions

were matched with the position of peaks observed in the experimental data. These set of angles were then used as θ_i and fitted with least squares method, which gave their respective weights w_i. Consequently, five prominent peaks were found sufficient to match the experimental data with an standard error of 0.0025. The authors have assumed a positive constant normalized weight fraction (between 0 and 1) for each angle (θ_i) such that the total weight sums up to 1.

Elemental mapping. Energy-dispersive X-ray spectroscopy **(**EDX) was performed with a JEOL 7600G SEM FEG.

Tensile tests. The mechanical properties of the assemblies were evaluated using a Universal Mechanical Tester (UMT-3, Bruker). The load sensor has a resolution of 0.5 mN and is able to accurately measure forces between 0.1 and 10 N. The specimens were cut in 20 mm long and 5 mm wide rectangles prior to the mechanical evaluation. The loading rate was 5 µm/s. An optical image with the specimen after tensile testing is shown in Figure S8b. An average of two measurements per assembly was performed. The consistency of the results is confirmed by the low error rates, as listed in Table S1. Young's modulus was extracted from the linear region of the stress-strain curves corresponding to the 0.16-0.40% strain range.

Experimental details

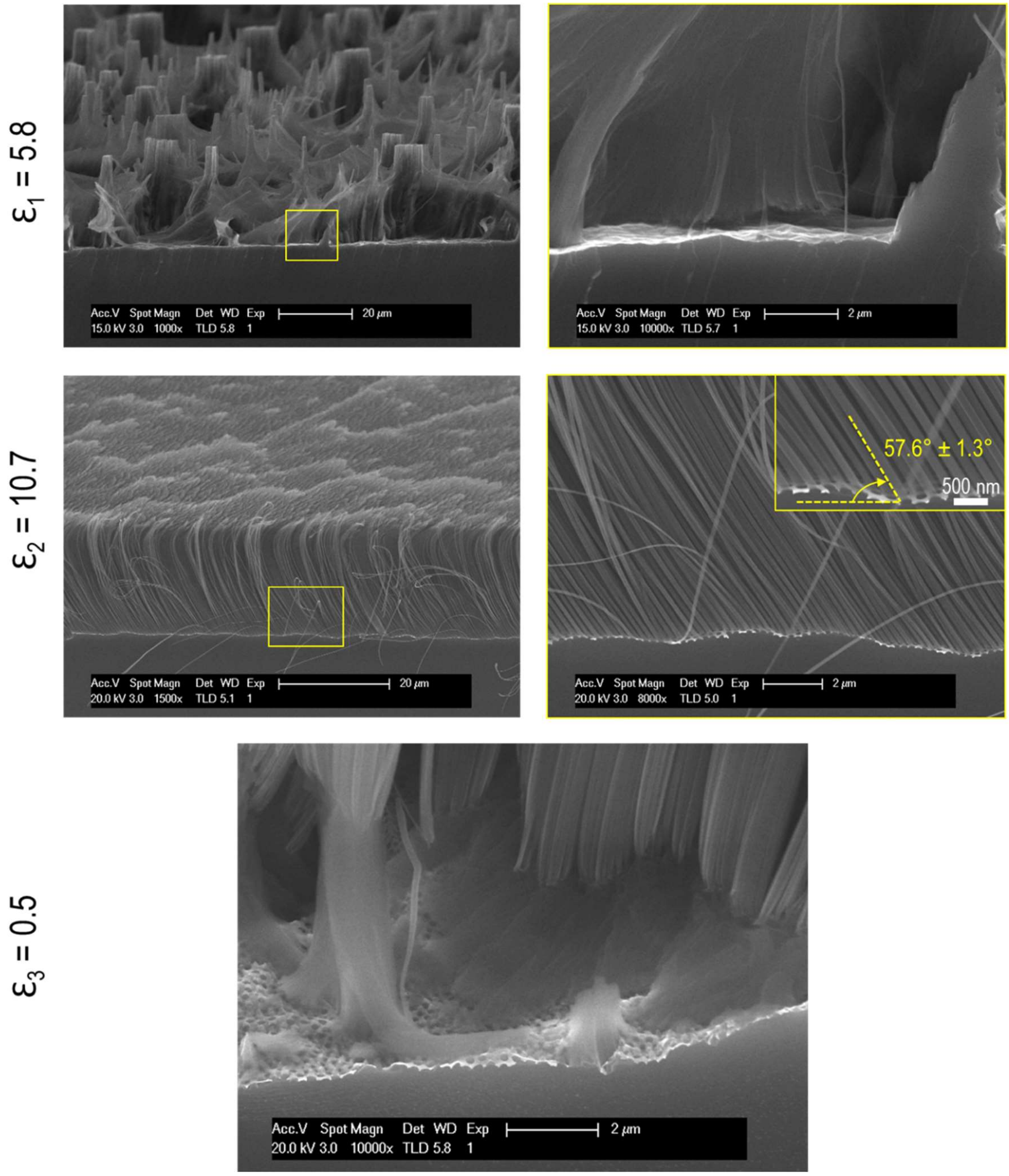

Figure S1. Silicon etching under different conditions. Etching in a solution with ε_1 = 5.8 results in the formation of porous SiNWs, while the etching in a solution with ε_2 = 10.7 follows non-<100> directions. The measured angle indicates <111> -oriented SiNWs. The etching time for both solutions was 20 minutes. For the high oxidant concentration, ε_3 = 0.5, highly porous SiNWs are formed.

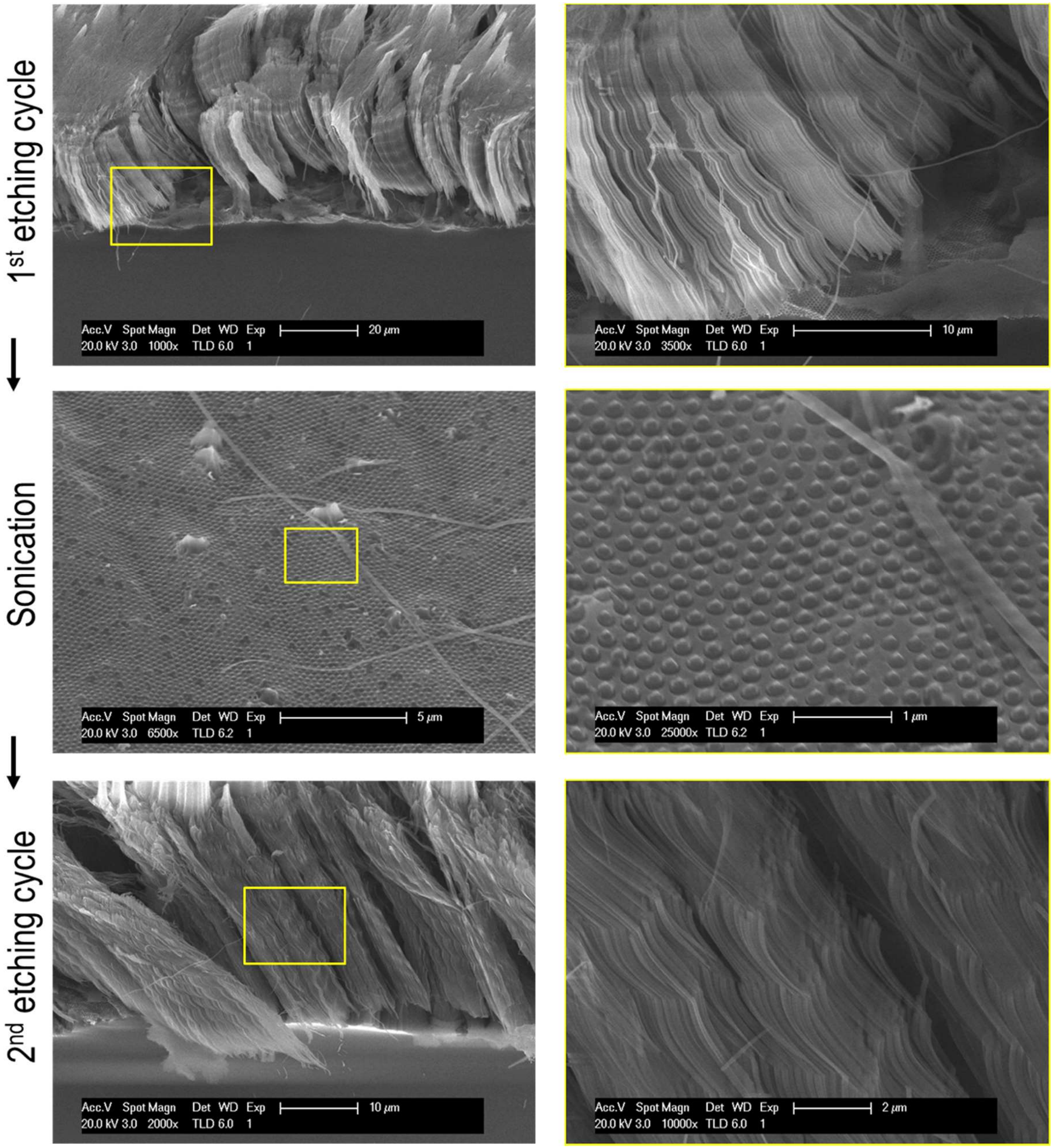

Figure S2. Details on the continuous synthesis of k-SiNWs sustained by chemical peeling. A porous segment is induced at the base of the k-SiNWs to allow the separation of the k-SiNWs from their Si substrate by changing the etching conditions. The k-SiNWs can be collected with a short ultrasonic treatment. The metal mask can be reused for a new etching sequence. It is important to note that after the ultrasonic treatment etching defects will remain on the substrate and the low ε etching conditions are expected to affect only the k-SiNWs. The etching will proceed normally in the areas with intact metal mask. This is an interesting advantage of this selective method as it facilitates the extraction of only nanowires and not of the inherent defects.

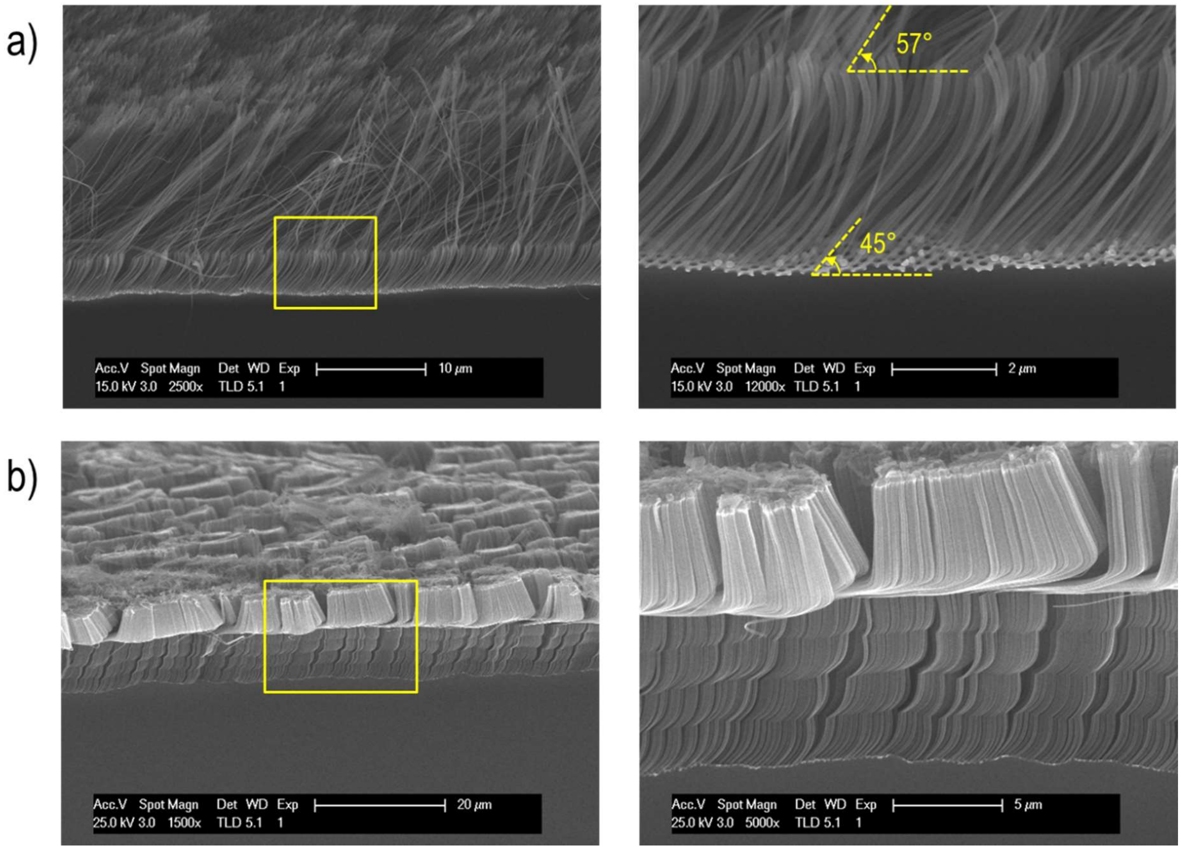

Figure S3. Customized Si-nanostructures by metal assisted chemical etching. 15 minutes etching in a ε_2=10.7 solution followed by a 1 minute quench in methanol and another 5 minutes etching results in the etching of Si nanostructures as shown in panel (a). Other nanostructures can be etched by alternating 5 minutes or 3 minutes etch-quench sequences as shown in panel (b) for 2 consecutive 5 minutes etch-quench sequences followed by 4 consecutive 3 minutes etch-quench sequences. These types of nanostructures are too brittle for further processing as anode materials.

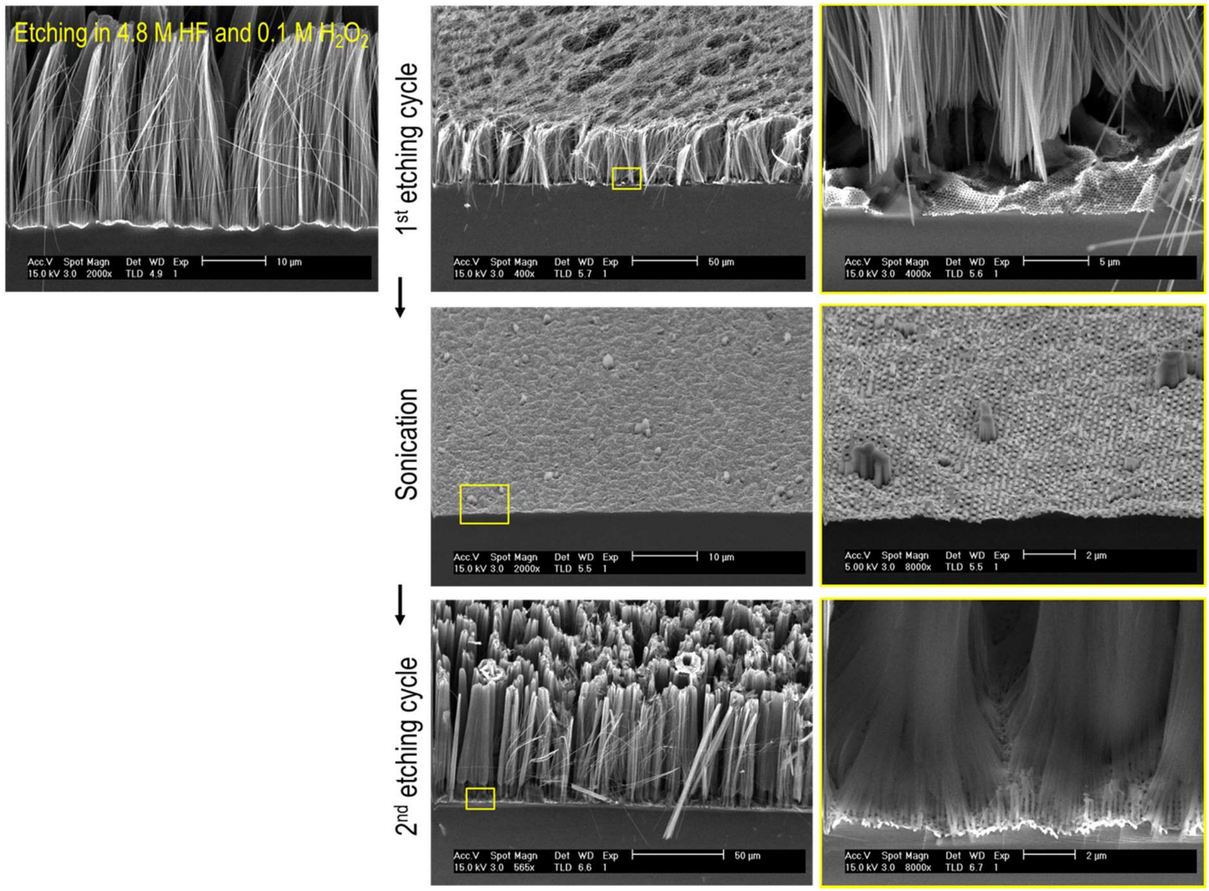

Figure S4. **Continuous SiNWs synthesis enabled by chemical peeling.** 50 µm straight SiNWs were produced by metal assisted chemical etching in a solution containing 4.8M HF and 0.1M H_2O_2 for a total etching time of 2 hours. The SiNWs are collected in methanol using a similar chemical peeling step that requires increasing of the concentration of the oxidizing agent by a factor of 10. Following the extraction of the SiNWs, the Au-patterned Si substrate can be reused for a new etching sequence.

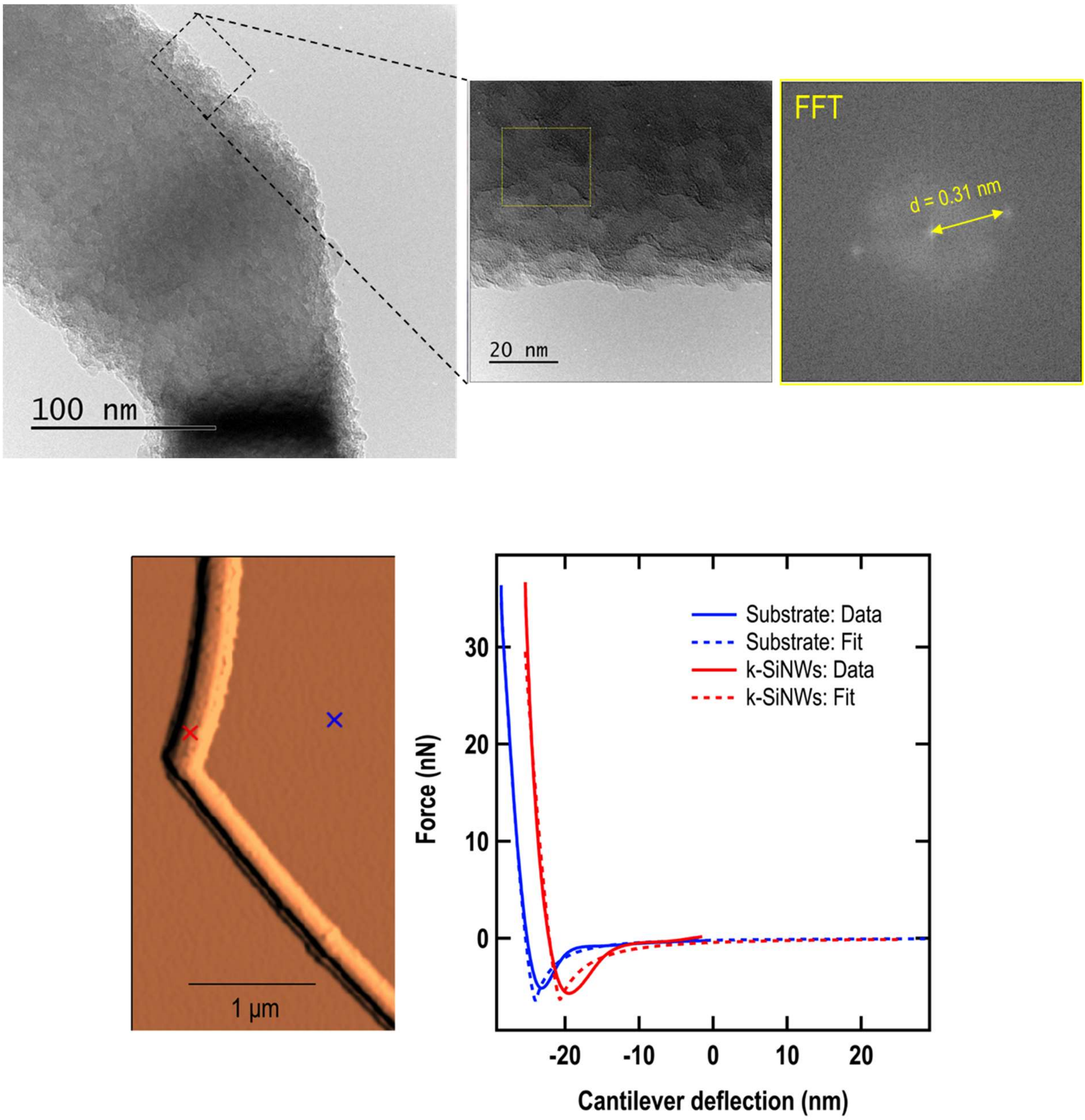

Figure S5. TEM and ImAFM results. (Top panels) TEM investigation of the joints of the k-SiNWs as well as the corresponding fast Fourier transform (FFT) pattern revealing an interplanar spacing d = 0.31 nm corresponding to the <111> orientation. (Bottom panels) For ImAFM studies, k-SiNWs were deposited on an oxidized Si substrate (*p*-type, <100>) and tip-surface forces were acquired to map their effective elastic modulus.

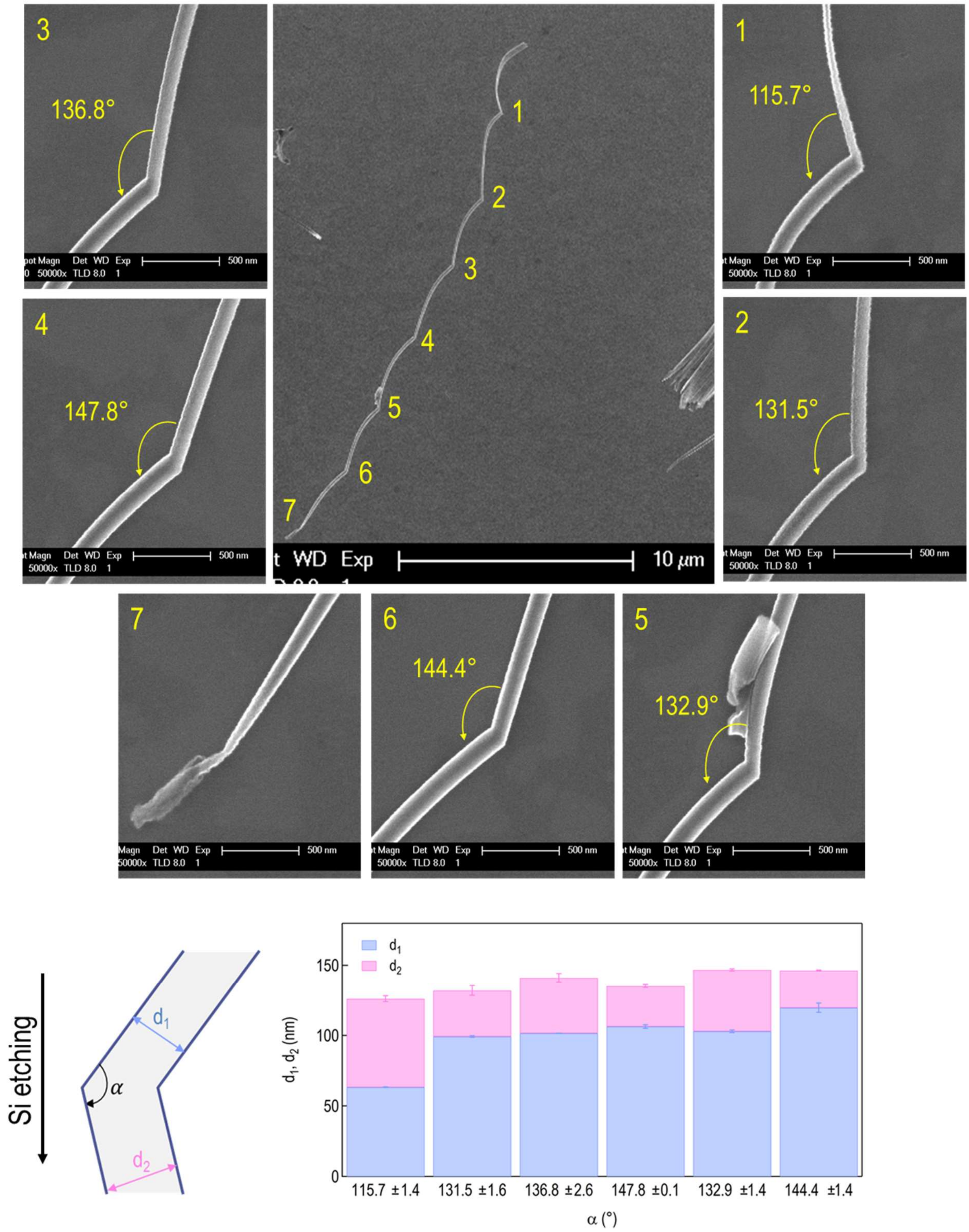

Figure S6. Scanning electron microscopy (SEM) characterization. For the statistical analysis of α, the kink angle formed by adjacent segments, k-SiNWs were imaged by SEM. Details of such joints can be observed in the top panels 1 - 6. In the bottom panel, the d_1 and d_2 diameters (schematically shown for a k-SiNW) are plotted as a function of α.

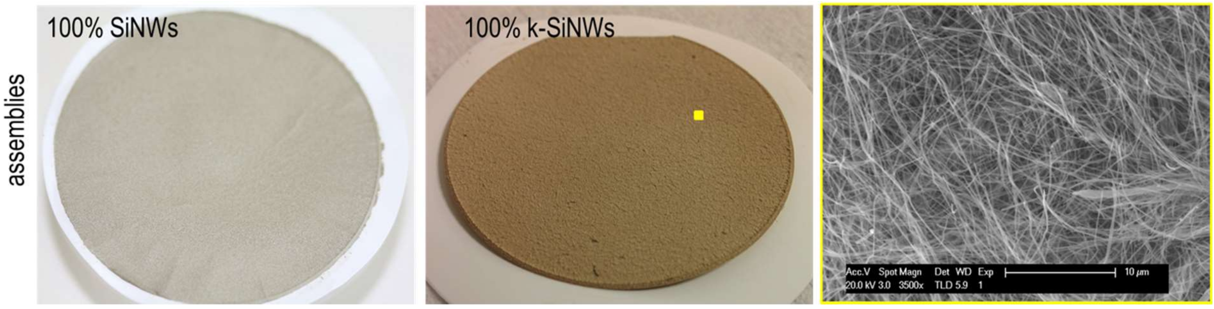

Figure S7. 100% SiNWs vs 100% k-SiNWs assemblies. 50 µm straight SiNWs were produced by metal assisted chemical etching as discussed in Figure S4. The k-SiNWs are fabricated as discussed in the previous section. The 100% SiNWs assembly is quite fragile and additional tools are required to separate it from the supporting filter, usually resulting in damage of the material. On the other hand, the 100% k-SiNWs assemblies provide sufficient mechanical strength to be easily manipulated due to the tendency to bundle of the k-SiNWs.

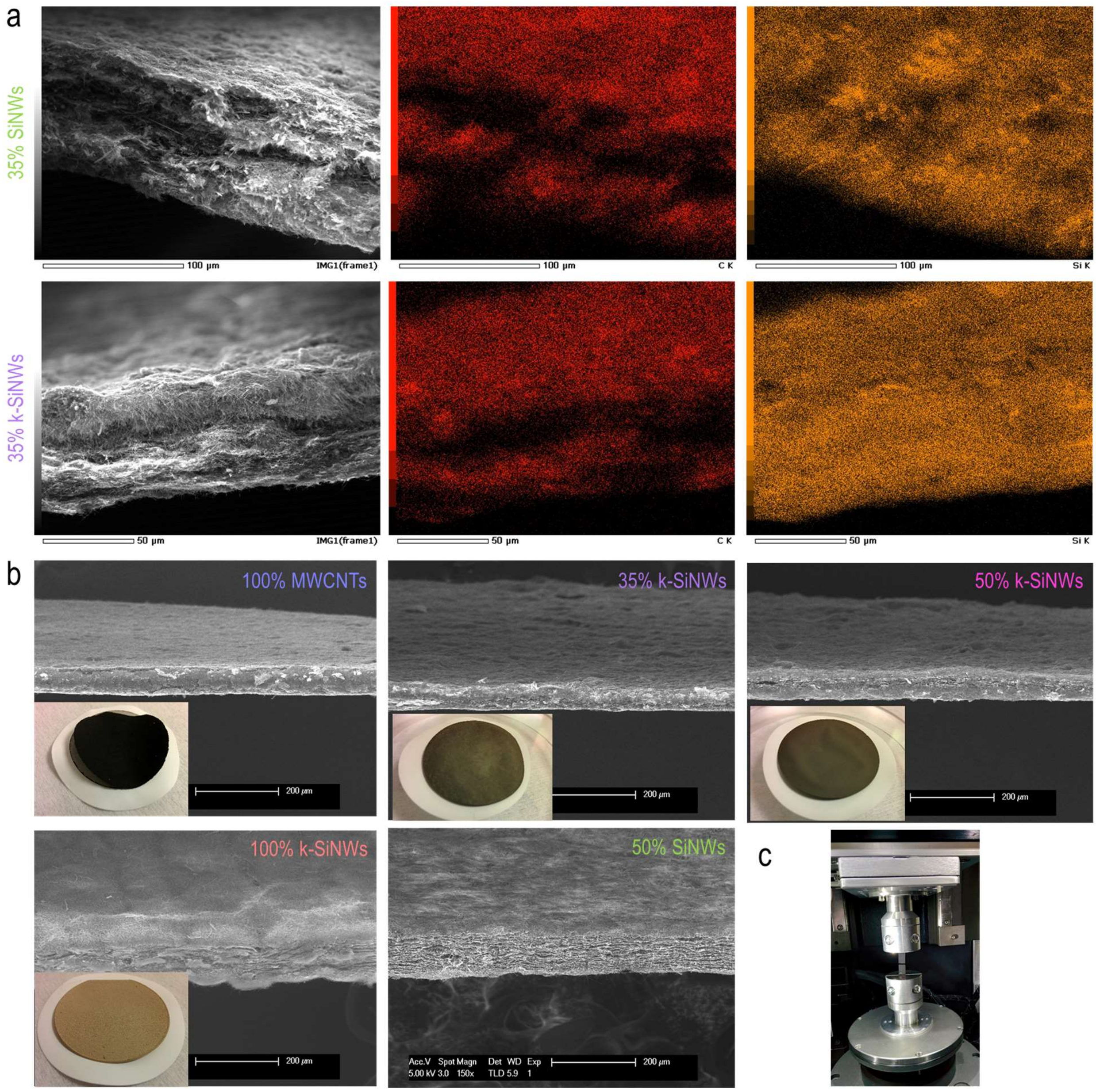

Figure S8. Nanostructured Si-based assemblies. Elemental mapping by energy-dispersive X-ray spectroscopy (a). SEM and optical images of the evaluated assemblies (b) as well as an optical image with the tensile testing setup (c).

Specimen	Thickness (μm)	Tensile strength (MPa)	Young's Modulus (GPa)	Apparent Density (g/cm^3)	Porosity (Φ)
100% MWCNTs (20 mg)	61.55 ±4.22	1.68 ±0.06	0.25 ±0.01	0.34 ±0.02	0.84 ±0.010
100% k-SiNWs (21.3 mg)	107.12 ±10.47	0.48 ±0.01	0.08 ±0.02	0.20 ±0.02	0.90 ±0.009
35% k-SiNWs (13.5 mg)	52.68 ±2.44	3.10 ±0.14	0.39 ±0.02	0.28 ±0.01	0.87 ±0.005
50% k-SiNWs (15 mg)	55.18 ±2.83	4.00 ±0.11	0.41 ±0.01	0.30 ±0.01	0.86 ±0.007
50% SiNWs (16.5 mg)	97.5 ±5.6	1.14 ±0.08	0.13 ±0.01	0.17 ±0.01	0.91 ±0.004

The porosity is calculated as follows:

$$\Phi = \frac{V_{Void}}{V_{Total}} = 1 - \frac{1}{V_{Total}}\left(\frac{m_{Si}}{\rho_{Si\,bulk}} + \frac{m_{MWCNTs}}{\rho_{C\,bulk}}\right),$$

where $\rho_{Si\,bulk}$= 2.33 g/cm^3 and $\rho_{C\,bulk}$= 2.26 g/cm^3.

Table S1. Physical parameters of the evaluated assemblies.

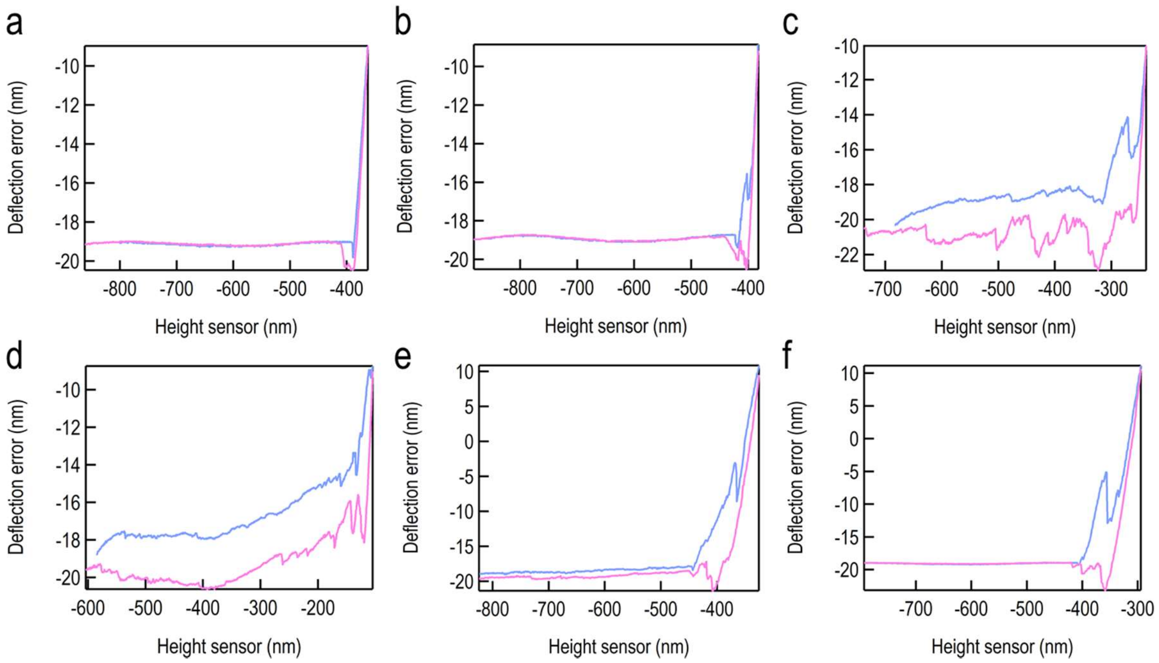

Figure S9. Force-distance curves taken at the same k-SiNW kink location on a pristine 35% k-SiNWs-based anode at different time intervals. The panels recorded from (a) to (f) reveal that the force-distance curves not only vary dramatically during the approach and retract motion of the cantilever sensor (blue and pink traces), they are also not similar from one to the other. Data indicates that the electrodes are displaying a sponge-like surface. AFM mechanical property measurements are based on contact mechanics assuming an elastic deformation of a sample surface. In our case, the sample is a porous, inter-twinned composite of kinked silicon nanowires with carbon nanotubes and the local deformation varies with time when identical measurements are taken at the same SiNW kink location (with 1 s time lapse).

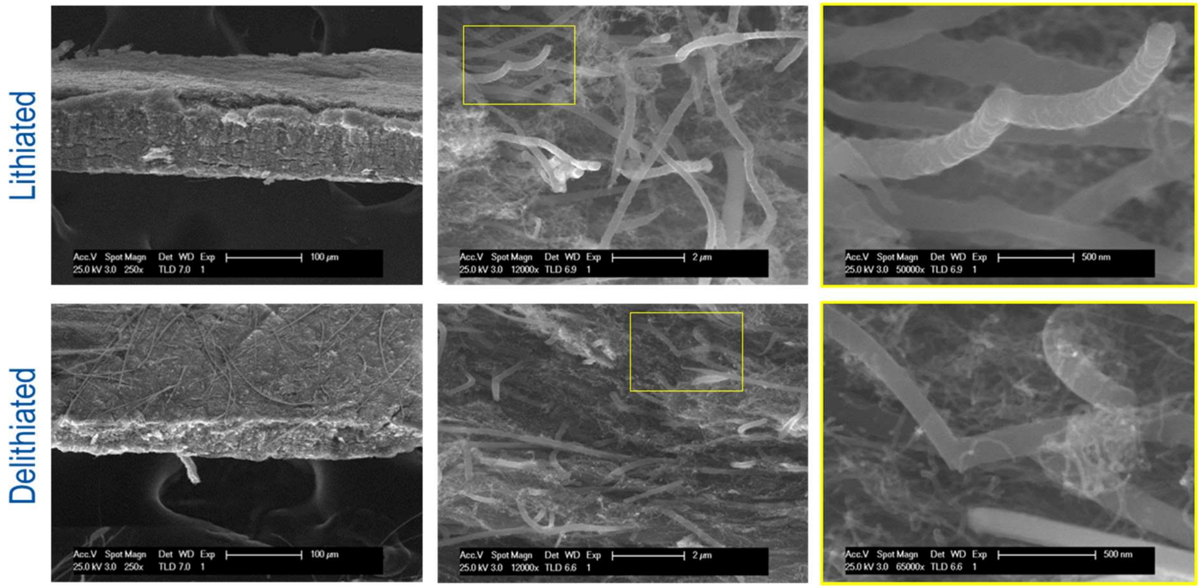

Figure S10. Morphology evolution of the 35% k-SiNWs-based anode assemblies after full lithiation and full delithiation. The lithiation of the k-SiNWs-based electrodes is realized by linear sweep voltammetry techniques, where the voltage is ramped down from the open circuit voltage of the cell to 10 mV at a rate of 0.1 mV/sec and held at this potential for 10 hours. The cell is further disassembled in an Ar-filled glove box, followed by a wash in acetonitrile prior to the SEM investigation. The k-SiNWs appear to sustain the high Li intake. Similarly, the delithiated samples are first lithiated and then delithiated by ramping up the voltage from 10 mV up to 2 V at a rate of 0.1 mV/sec. The cell is further disassembled in the air, followed by a wash in acetonitrile prior to the SEM investigation. The k-SiNWs seem restored to their initial shape.

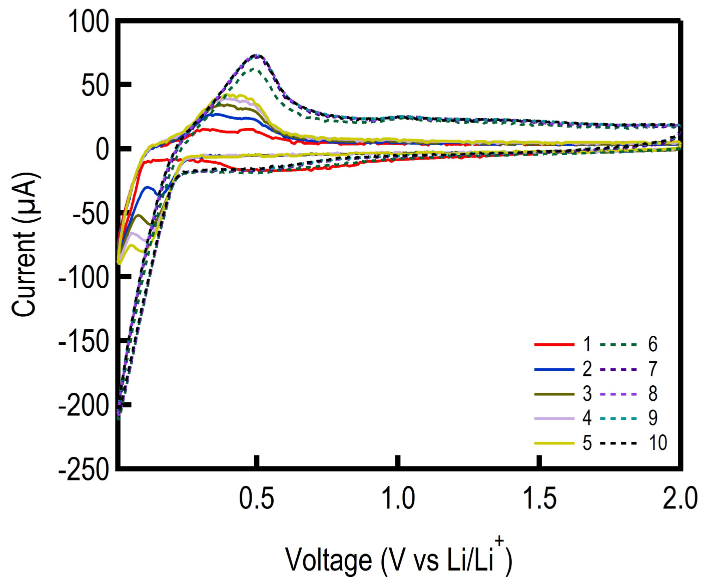

Figure S11. Cycling voltammetry. Cycling voltammetry profiles of the 35% k-SiNWs-based anode assembly. The scanning speed was 0.1 mV/s for the first 5 cycles and 1 mV/s for the following 5 cycles.

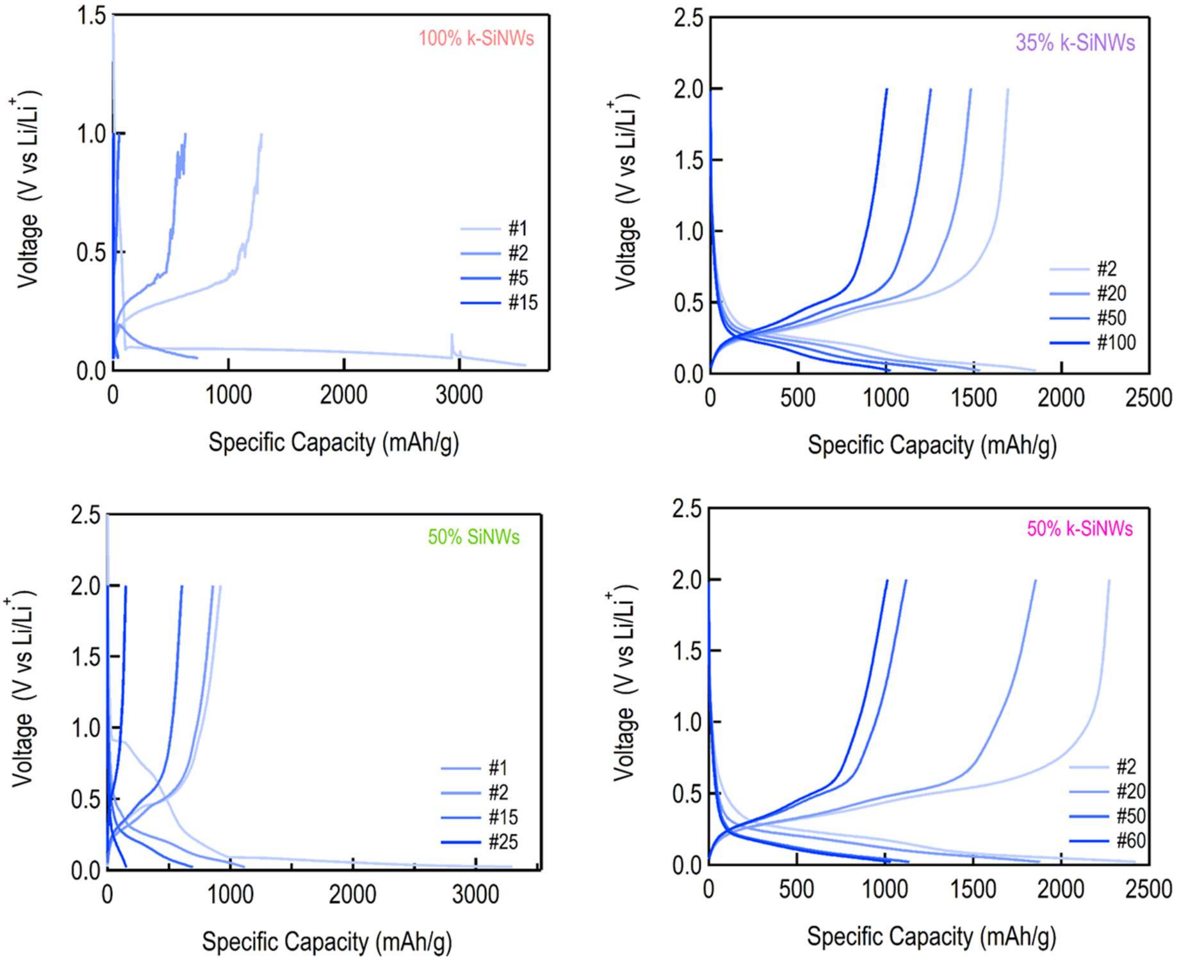

Figure S12. Electrochemical cycling tests of nanostructured Si-based assemblies. Galvanostatic charge-discharge profiles at different cycling extent of the nanostructured Si-based assemblies. The cycling rate is C/15, where 1C corresponds to 3600 mA/g.

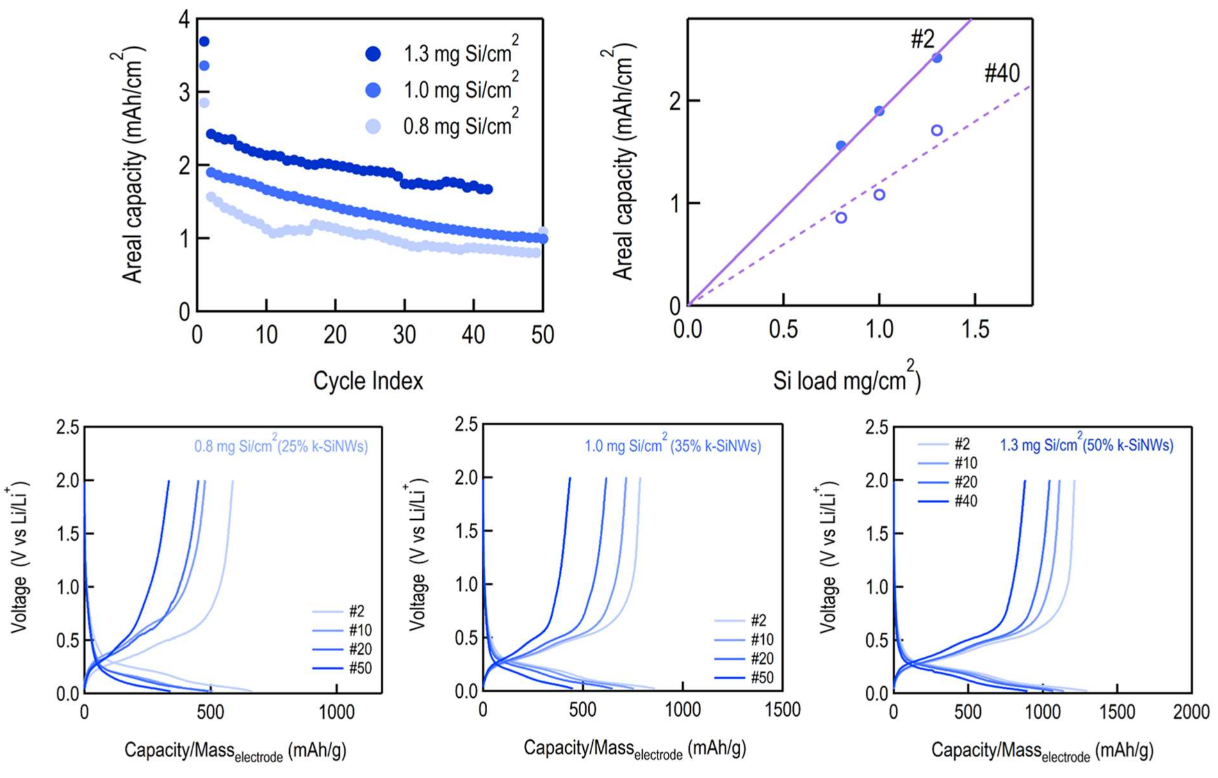

Figure S13. Areal capacity vs. gravimetric capacity. Electrochemical performance of k-SiNWs electrodes with different Si loadings. The areal capacity can linearly be increased with the Si loading, achieving 2.42 mAh/cm^2 for the assembly with 1.3 mg_{Si}/cm^2 cycled at 0.12 A/g (0.14 mA/cm^2). We also observe such dependence in the capacity fading, indicating invariability in the underlying degradation mechanisms. The corresponding galvanostatic charge-discharge curves are normalized with the total electrode mass (both Si and MWCNTs contributions).

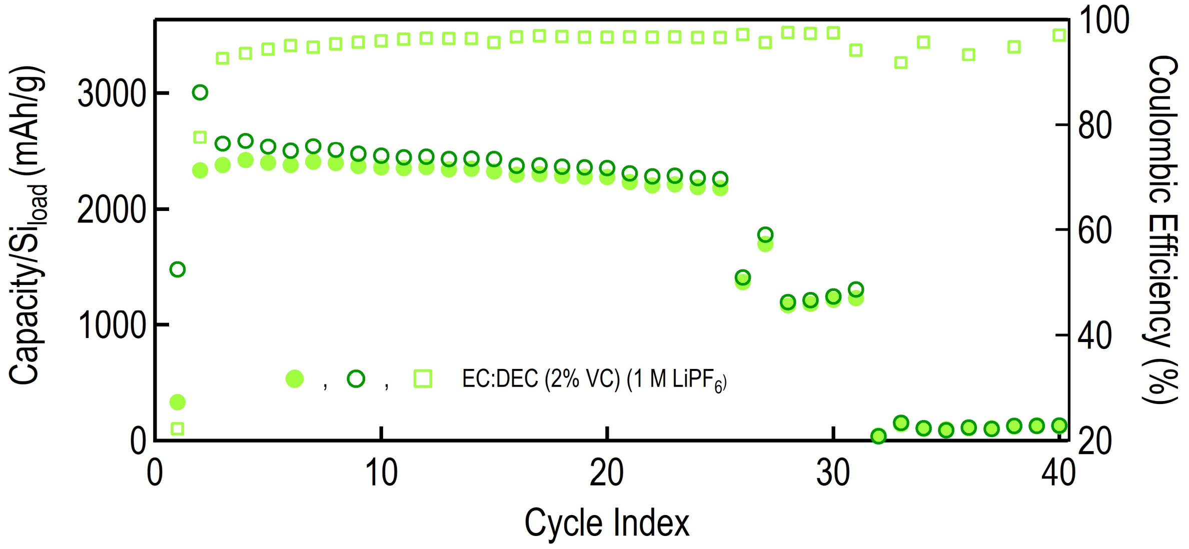

Figure S14. 35% k-SiNWs-based assembly cycling in conventional electrolytes. Specific capacity (left axis) and Coulombic efficiency (right axis) vs cycle index for conventional electrolytes for a pristine k-SiNWs-based electrode. On the left axis, open circles correspond to discharging and closed circles represent charging. Cycling was carried out between 0.02 to 2 V vs Li/Li^+ at a rate of C/15. For the k-SiNWs-based anode assembly, although the first 25 cycles are relatively stable, the capacity quickly fades as the cycling proceeds. The constant SEI development during Li insertion eventually causes shedding of the pre-formed SEI, exposing the untainted Si and sustaining thus the formation of new SEI layers.

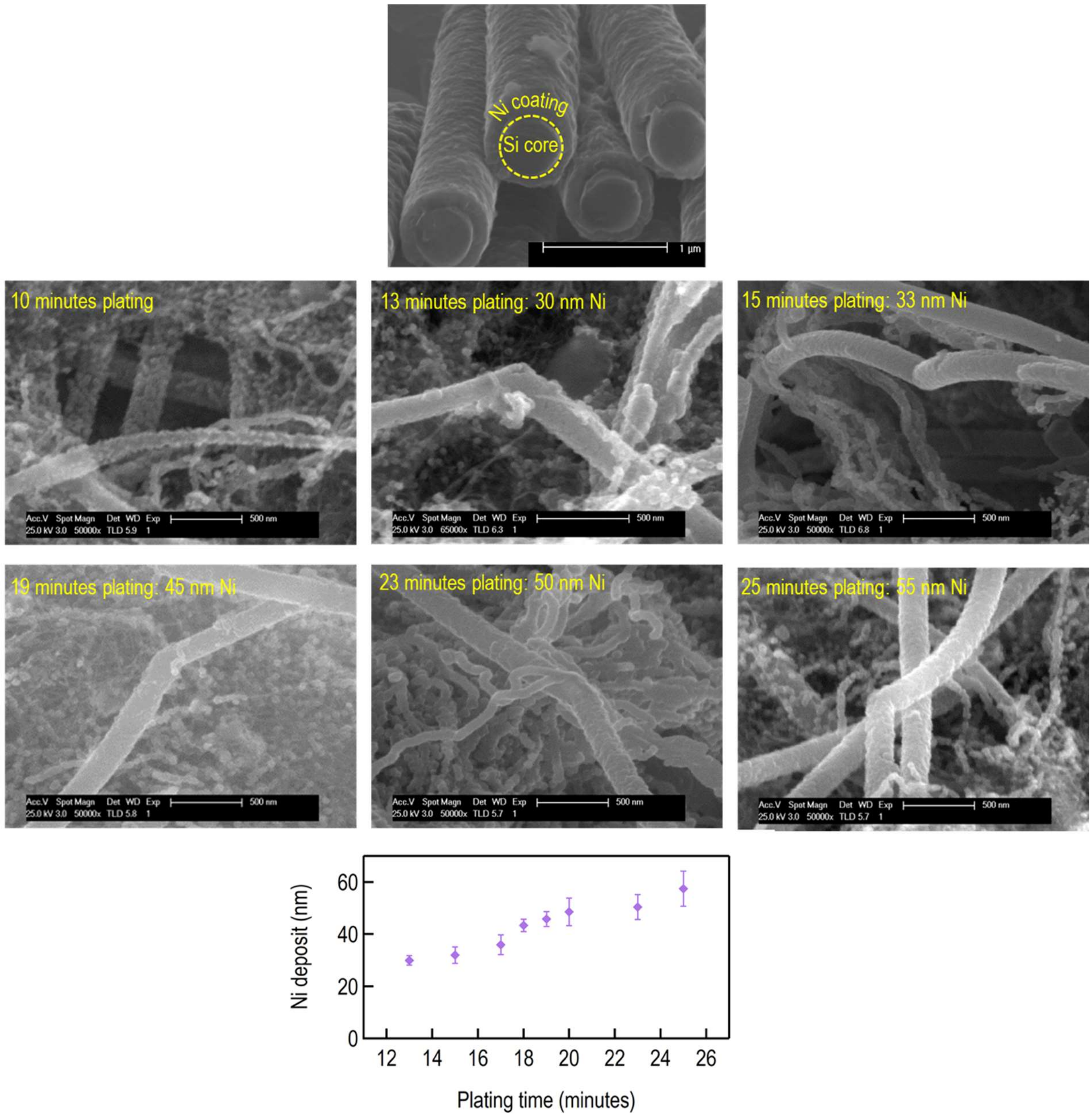

Figure S15. Characterization of Ni deposits. (Top panel) We developed the electroless coating procedure on thick SiNWs pillars as described in Refs. 1 and 7. (Bottom panels) For the k-SiNWs assemblies, continuous, thin Ni layers cannot be obtained easily. After 10 minutes in the plating bath, a discontinuous, porous Ni shell decorates the k-SiNWs. Longer times are required for continuous, conformal Ni coatings. The thickness of the Ni deposit was estimated by measuring the diameter of individual Ni-coated k-SiNWs using SEM.

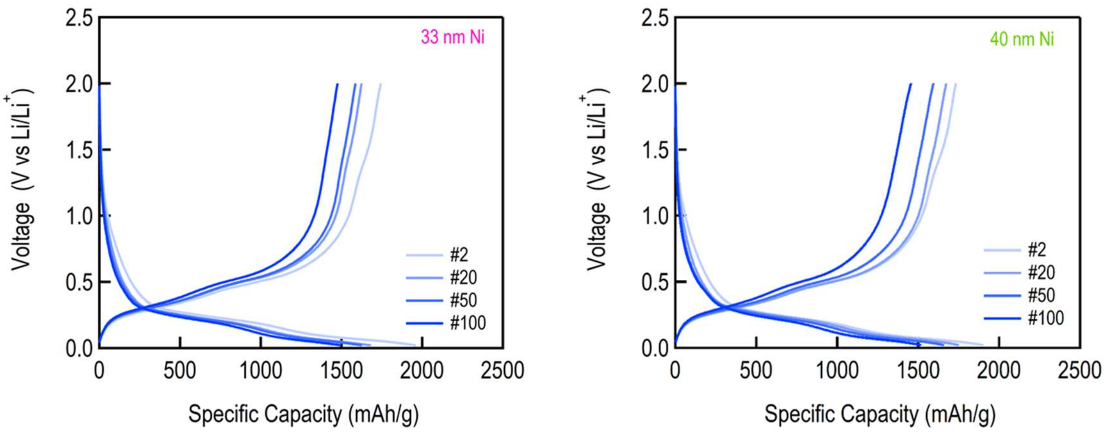

Figure S16. Galvanostatic charge-discharge of 35% k-SiNWs assembly with different Ni coating thicknesses. (Left panel) The Ni thickness is 33 nm. (Right panel) The Ni thickness is 40 nm. The Si load is 1 mg_{Si}/cm^2, while the cycling rate is C/15.

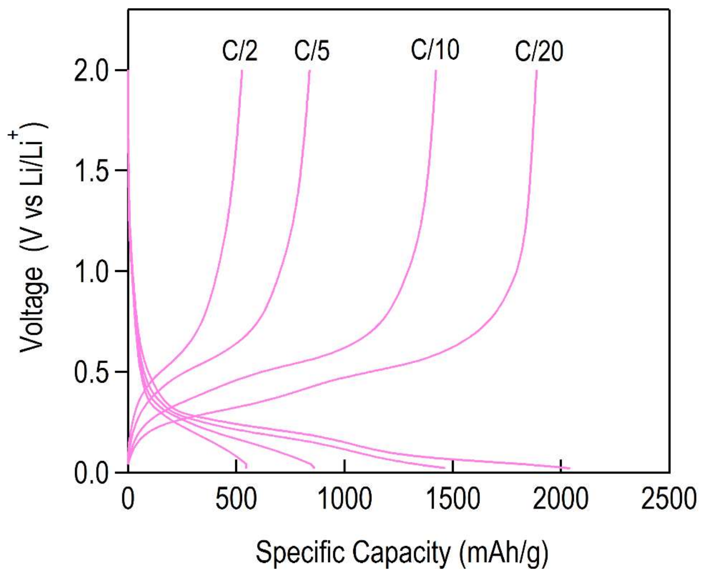

Figure S17. Galvanostatic charge-discharge profiles at different C rates. Data is taken on the as-prepared 35% k-SiNWs-based anode assembly.

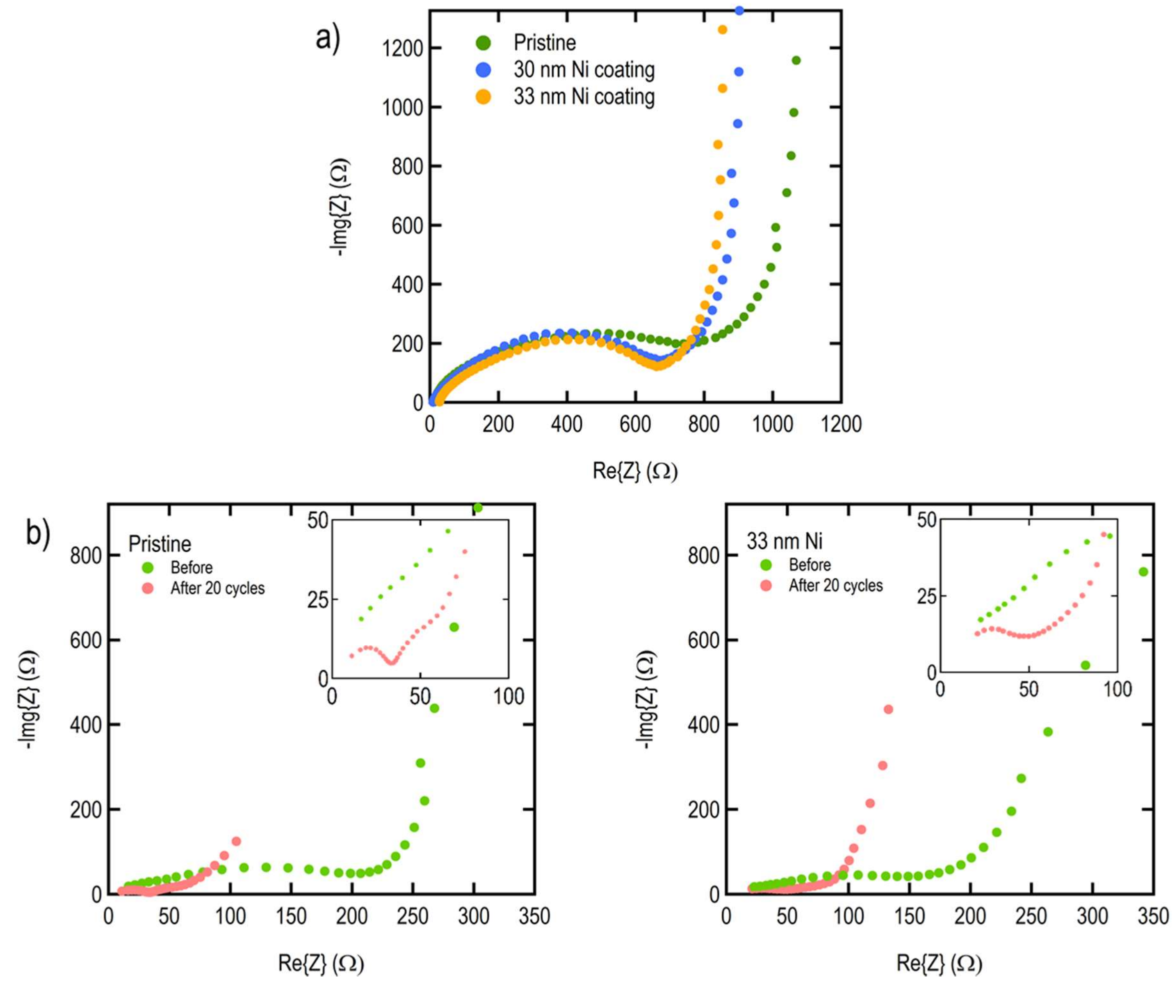

Figure S18. Electrochemical impedance characterization of pristine and Ni-coated 35% k-SiNWs anode assemblies. Electrochemical impedance plots for the as-assembled half cells suggesting that the 33 nm thick Ni shell provides the lowest resistance for the Li transport (a). Comparison of electrochemical impedance spectra before and after 20 cycles for pristine and 33 nm thick Ni-coated k-SiNWs anode assemblies probed in half cells (b).

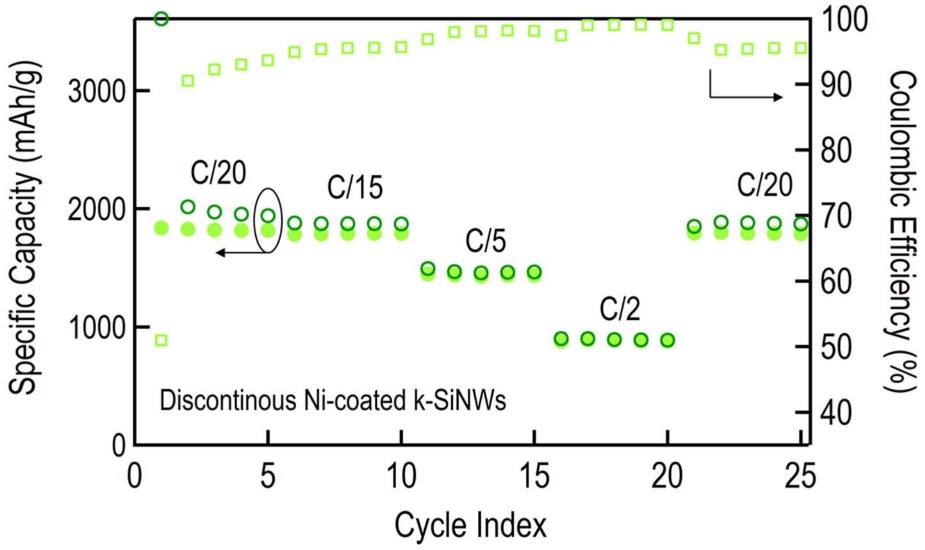

Figure S19. Rate capability of discontinuous, porous Ni-coated 35% k-SiNWs anode assemblies. Specific capacity (left axis) and Coulombic efficiency (right axis) vs cycle index for a discontinuous, porous Ni-coated k-SiNWs electrode. On the left axis, open circles correspond to discharging and closed circles represent charging.

References:

1. Lancaster, J. R. *et al.* Toward a Universal Method To Pattern Metals on a Polymer. *Chem. Mater.* **20,** 6583–6585 (2008).
2. Platz, D., Tholén, E. A., Pesen, D. & Haviland, D. B. Intermodulation atomic force microscopy. *Appl. Phys. Lett.* **92,** 153106 (2008).
3. Platz, D., Forchheimer, D., Tholén, E. A. & Haviland, D. B. The role of nonlinear dynamics in quantitative atomic force microscopy. *Nanotechnology* **23,** 265705 (2012).
4. Platz, D., Forchheimer, D., Tholén, E. A. & Haviland, D. B. Interaction imaging with amplitude-dependence force spectroscopy. *Nat. Commun.* **4,** 1360 (2013).
5. Platz, D., Forchheimer, D., Tholén, E. A. & Haviland, D. B. Interpreting motion and force for narrow-band intermodulation atomic force microscopy. *Beilstein J. Nanotechnol.* **4,** 45–56 (2013).
6. Forchheimer, D., Platz, D., Tholén, E. A. & Haviland, D. B. Model-based extraction of material properties in multifrequency atomic force microscopy. *Phys. Rev. B* **85,** 195449 (2012).
7. Sandu, G. *et al.* Surface Coating Mediated Swelling and Fracture of Silicon Nanowires during Lithiation. *ACS Nano* **8,** 9427–9436 (2014).
